# Uncertainty quantification of the lattice Boltzmann method focussing on studies of human-scale vascular blood flow

**DOI:** 10.1038/s41598-024-61708-w

**Published:** 2024-05-17

**Authors:** Jon W. S. McCullough, Peter V. Coveney

**Affiliations:** 1 Centre for Computational Science, Department of Chemistry, University College London, London, UK; 2 Centre for Advanced Research Computing, University College London, London, UK; 3 Informatics Institute, University of Amsterdam, Amsterdam, Netherlands

**Keywords:** Lattice Boltzmann method, Uncertainty quantification, Blood flow simulation, Biomedical engineering, Fluid dynamics

## Abstract

Uncertainty quantification is becoming a key tool to ensure that numerical models can be sufficiently trusted to be used in domains such as medical device design. Demonstration of how input parameters impact the quantities of interest generated by any numerical model is essential to understanding the limits of its reliability. With the lattice Boltzmann method now a widely used approach for computational fluid dynamics, building greater understanding of its numerical uncertainty characteristics will support its further use in science and industry. In this study we apply an in-depth uncertainty quantification study of the lattice Boltzmann method in a canonical bifurcating geometry that is representative of the vascular junctions present in arterial and venous domains. These campaigns examine how quantities of interest—pressure and velocity along the central axes of the bifurcation—are influenced by the algorithmic parameters of the lattice Boltzmann method and the parameters controlling the values imposed at inlet velocity and outlet pressure boundary conditions. We also conduct a similar campaign on a set of personalised vessels to further illustrate the application of these techniques. Our work provides insights into how input parameters and boundary conditions impact the velocity and pressure distributions calculated in a simulation and can guide the choices of such values when applied to vascular studies of patient specific geometries. We observe that, from an algorithmic perspective, the number of time steps and the size of the grid spacing are the most influential parameters. When considering the influence of boundary conditions, we note that the magnitude of the inlet velocity and the mean pressure applied within sinusoidal pressure outlets have the greatest impact on output quantities of interest. We also observe that, when comparing the magnitude of variation imposed in the input parameters with that observed in the output quantities, this variability is particularly magnified when the input velocity is altered. This study also demonstrates how open-source toolkits for validation, verification and uncertainty quantification can be applied to numerical models deployed on high-performance computers without the need for modifying the simulation code itself. Such an ability is key to the more widespread adoption of the analysis of uncertainty in numerical models by significantly reducing the complexity of their execution and analysis.

## Introduction

As numerical methods become an ever more intrinsic part of the process of scientific and engineering research and development, trust in such models can be increased through explicit verification, validation and uncertainty quantification processes (collectively, VVUQ). Such trust is essential in fields such as the medical domain where the end users of models traditionally have less of a foundation in numerical methods and high-performance computing. In light of this, regulators are often requiring VVUQ as a component of the approval process where software solutions are used in medical device design, diagnosis and analysis^1–4^.

The three elements of VVUQ focus on addressing different components of building trust in a model. Verification ensures that the model captures the behaviour of the fundamental equations of the scenario of interest. In a fluid mechanics setting, this may involve demonstration that the solver can replicate analytical flow solutions such as Poiseuille flow through a cylindrical pipe. Validation seeks to demonstrate whether the simulation is able to represent the physical reality it purports to model. This will typically involve comparison between the numerical results and those obtained in a controlled experiment or observation. In some settings, validation may alternatively be achieved through comparison to a ‘gold standard’ numerical solution or method that is regarded as ‘exact’ to a meaningful extent. This step is often more difficult to achieve than verification due to the broad range of factors that may impact reality which cannot be feasibly resolved in a simulation. Finally, the focus of uncertainty quantification is to quantify where errors may be generated in the model, how they may propagate and what impact they have on the accuracy of the simulations. These uncertainties can be split into two broad categories. Epistemic uncertainties are those that are systemic to the model and may arise from the theoretical representation of the scenario of interest not being adequately captured. From this perspective, the very construction of any model requires some uncertainties to be adopted as a result of approximations made in its development. Aleatoric uncertainties however are those that arise due to uncertainty in the model itself. Such an example may be found in the random seeds used to initialise particles within a molecular dynamics simulation^5,6^.

VVUQ, and in particular uncertainty quantification, can be applied to both a generic simulation technique or the study of a specific situation. This latter case may be conducted to demonstrate how a model may be used in the diagnosis or risk analysis of a medical condition based on biomarkers. A recent discussion of the challenges in applying VVUQ to a range of computational methods is provided in a recent theme issue introduced by Coveney et al.^7^. In this particular study, we will be focussing on the former situation with an emphasis on fluid simulation with the lattice Boltzmann method (LBM). We will use the LBM to study flow in a bifurcating geometry that is representative of the vascular junctions found in the arterial and venous networks of the human body. We conduct an uncertainty study of flow in these domains through a systematic variation of LBM simulation parameters and boundary condition values. Here, we identify the most influential parameters and how they impact the macroscopic output of the simulations. We also extend this study to the simulation of patient specific vessels with similar bifurcating characteristics to demonstrate how the techniques can be applied to more realistic cases.

A challenge in conducting thorough uncertainty quantification studies is the number of simulations needing to be conducted expanding as further input parameters are considered. The number of simulations to be conducted in a campaign can be determined by raising the order of the conducted analysis (plus 1) to the number of input parameters. The rapid growth caused by this power law behaviour represents the so-called ‘curse of dimensionality’ of uncertainty quantification studies. Unless simulations execute extremely quickly at low compute cost, this quantity can become prohibitive to execute even on powerful high-performance computing resources. For the cases studied in the current work, the number of model executions was manageable within the compute resources available. Within the LBM more generally however, a full study of the algorithm and its various implementations may become untenable when using conventional approaches. For example, if a multiple relaxation time collision kernel were used to solve a 3D simulation, it would require up to 27 input variables just to describe this process without considering other variables related to discretisation, simulation time or boundary conditions. Techniques have been developed within the VVUQ literature to reduce the burden of the curse of dimensionality^8^. One option may be to utilise a cheaply evaluated surrogate to represent the more complex numerical model. Another option would be to utilise techniques such as dimension-adaptive sampling that help to guide the choice of input parameter value such that the most effort is invested in changing the most influential inputs^9,10^. A further approach is active subspace methods where, for example, a neural network is used to develop a surrogate model representing the more detailed calculation^8,11,12^. Kernel-based methods are also available to assist in dimension reduction^13^. Edeling^12^ gives examples for deep active subspace methods being used to study problems with dozens of input parameters in epidemiological scenarios. Such approaches have also been used successfully in problems with hundreds of parameters in molecular dynamics^14^. In the current study, we have limited our input parameter space (discussed further in Methods) to a relatively select set of values.

With increasing computational power, many numerical models may be seen to be increasing in complexity with more input parameters. In the biomedical space, this may be further expanded by the use of patient specific input data that may provide dozens or hundreds of tunable parameters (e.g. in a large vascular domain). Demands for VVUQ of such *in silico* models by regulatory bodies will continue to push the need for large scale computers to be developed to assess such characteristics. Because of the demand for many hundreds or thousands of simulations to be run to conduct VVUQ studies, this remains a strong motivating factor for the use of exascale computers capable of $10^{18}$ floating point operations per second to expedite such work.

In Methods we introduce the numerical codes that have been used in this study for both the simulations and the uncertainty analysis. TheResults section will present the observed findings from our uncertainty studies and these will be expanded upon in Discussion. The findings of our study will be summarised in the Conclusion section.

## Methods

**Figure 1:**
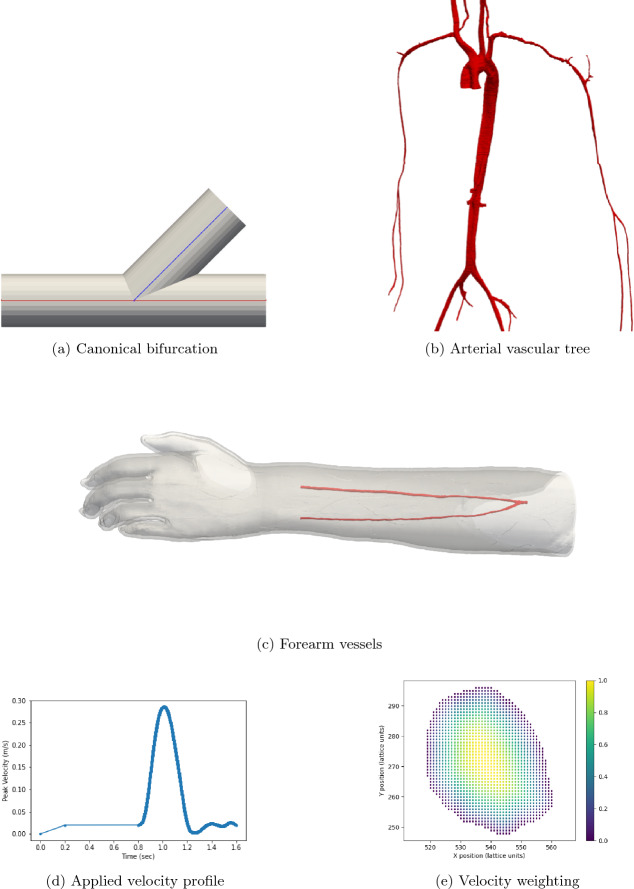
In this study, we have chosen an angled shape with circular cross-sections for the canonical bifurcation (**a**) to represent those found naturally within human-scale vascular networks. Several such cases can be identified within the arterial domain presented in (**b**). The bifurcations studied in this work have diameters of 1–10 mm. In our work, a constant velocity is applied to the inlet of the canonical bifurcation with a parabolic profile. The individualised vessels for the radial and ulnar arteries in the left forearm in (**c**) are used to illustrate how the UQ technique can be applied to patient specific domains. (**d**) and (**e**) represent the velocity conditions applied to the inlet (right-hand side vessel boundary) of the personalised forearm arteries. The transient maximum velocity applied over time, including the initialisation period, is indicated by (**d**). The spatial variation of the flow profile is imposed by scaling the maximum velocity of (**d**) by the local weight indicated by (**e**) to determine the velocity applied at that particular location. Though this approach allows free choice of the profile shape, we have chosen to implement a quasi-parabolic profile in this work.

Bifurcations are a characteristic feature of vascular blood flow networks at all length scales, from the aorta to capillary beds. To reflect this, we conduct uncertainty studies on an idealised version of a canonical bifurcation to represent the junctions that can be seen within human-scale vessels (Fig. 1).

In this study, we have used the open-source 3D fluid dynamics code HemeLB^15–21^ to solve the flow in the test geometries. HemeLB has been optimised to efficiently simulate the sparse domains characteristic of blood vessels and uses the LBM to solve for the macroscopic flow in such regions. The LBM algorithm stores mesoscopic variables in the form of local population distribution functions at a set of discrete grid points representing the fluid domain. These values are updated through time by a local collision operator and a streaming process that swaps values between neighbouring grid points. This approach solves a discretised form of the Boltzmann equation that can be demonstrated to replicate the Navier–Stokes equations for incompressible fluid flow for certain flow conditions, in particular a sufficiently low Mach number. The macroscopic fluid variables of pressure (proportional to density in the LBM scheme) and momentum can be found through the moments of the population distribution functions at each local grid point. Examples of these derivations and calculations can be found widely in the LBM literature^22–26^. Here, we expand the above outline to provide a brief description of the implementation of the LBM to highlight key factors to be investigated in our study. Within the LBM formulation in HemeLB, the 3D fluid domain is constructed from a set of evenly spaced grid points separated spatially in **x** by $\Delta x$. In this study, we use the single relaxation time operator to update the local population distribution functions, $f_i$, in time, *t*, where *i* represents the number of distributions stored at each site. We utilise the D3Q19 velocity set to require 19 such distributions. The set ${\textbf {c}}_i$ contains the vectors describing the motion required to move from the source node to itself or each of the 18 neighbouring sites considered in D3Q19 ($i = 1-6$ $\left[ \left( \pm 1, 0, 0 \right) ,\left( 0, \pm 1, 0 \right) ,\left( 0, 0, \pm 1 \right) \right)$ and $i = 7-18$ $\left[ \left( \pm 1, \pm 1, 0 \right) ,\left( \pm 1, 0, \pm 1 \right) ,\left( 0, \pm 1, \pm 1 \right) \right]$.) The equation,

1 $$\begin{aligned} f_i({\textbf {x}}+{\textbf {c}}_i \Delta t,t + \Delta t) = f_i({\textbf {x}},t) -\frac{\Delta t}{\tau }(f_i({\textbf {x}},t)-f_i^{eq}({\textbf {x}}, t)), \end{aligned}$$

updates the distributions based on their difference from the equilibrium distribution $f_i^{eq}$,

2 $$\begin{aligned} f_i^{eq}({\textbf {x}}, t) = w_i \rho ({\textbf {x}},t) \left( 1 + \frac{{\textbf {c}}_i\cdot {\textbf {u}}}{C_s^2} + \frac{({\textbf {c}}_i\cdot {\textbf {u}})^2}{C_s^4} - \frac{|{\textbf {u}}|^2 }{C_s^2}\right) . \end{aligned}$$

For D3Q19 the distribution weights, $w_i$, are 1/3 for $i = 0$ (staying at the site), 1/18 for $i = 1-6$ (streamed to nearest neighbours) and 1/36 for $i = 7-18$ (streamed to next nearest neighbours). $C_s$ represents the speed of sound of the fluid and evaluates to $\frac{1}{\sqrt{3}}$. The zeroth moment of $f_i$ determines the local density,

3 $$\begin{aligned} \rho ({\textbf {x}},t) = \sum _{i}f_i({\textbf {x}}, t), \end{aligned}$$

and the first moment determines momentum (and thus velocity **u**),

4 $$\begin{aligned} \rho ({\textbf {x}},t){\textbf {u}} = \sum _{i}f_i({\textbf {x}},t){\textbf {c}}_i. \end{aligned}$$

Pressure in this form of LBM is a scalar multiple of density $p({\textbf {x}},t) = C_s^2 \rho ({\textbf {x}}, t)$. The single relaxation parameter used in this model, $\tau$, has a known influence on the accuracy and stability of an LBM simulation and is related to the kinematic viscosity of the fluid and the discretisation parameters,

5 $$\begin{aligned} \nu = C_s^2 \left( \tau -\frac{1}{2}\right) \frac{\Delta x}{\Delta t}. \end{aligned}$$

For the LBM, the computational Mach number is defined as $Ma = \frac{{\textbf {u}}}{C_s}$.

Here we study the aforementioned bifurcation domain at two levels of spatial decomposition. We vary the LBM parameters of the simulation to illustrate the intrinsic impact of the algorithm itself on the numerical outcome. Additionally, we also investigate the effect of boundary condition choices on flow behaviour. The implementation of boundary conditions within HemeLB are described further in Nash et al.^19,^ but we will provide a brief summary of their implementation here for reference. Pressure boundary conditions are imposed such that mixed Dirichlet-Neumann conditions are enforced on the chosen surface: pressure being fixed to a chosen value, no velocity component parallel to the surface and no velocity gradient normal to the surface. This is imposed by constructing a ‘phantom site’ whose population distributions are constructed such that the desired values are streamed to the unknown distributions of the physical boundary site. A first-order finite difference approximation dictates that, to impose the assumed boundary behaviour, the velocity component present at the phantom site matches the velocity at the outlet site in the normal direction to the boundary surface and that the pressure of the phantom site is equal to that desired to be imposed on the boundary. The velocity condition is imposed through the method described by Ladd^27^. At the boundary surface, the sites are updated via the well-known simple bounceback method (where distributions crossing a wall boundary are reflected in the reverse direction) and then a correction is applied to each site to ensure that the desired fluid velocity at the site is imposed. This correction is calculated via $-2w_i \rho \textbf{u}\cdot \textbf{c}_i / C^2_s$. Solid walls are represented by the Bouzidi et al. boundary condition^19,28^. The implementation of this method in HemeLB imposes a linear interpolation between values of the distribution of interest as determined by the simple bounceback method and that at its target site.

For this study, the construction of the LBM within HemeLB defines the set of parameters being examined. The discretisation parameters of time, $\Delta t$, and space, $\Delta x$, govern the conversion of the simulation between the physical and dimensionless domains. Within the LBM, a key conversion between physical and dimensionless domains is the choice of the viscosity and density of the fluid being studied. In HemeLB, these values are held constant at 4 mPa.s and 1000 kg/m^3^, respectively, with an assumption of Newtonian fluid rheology. We take a macroscopic view of blood and, based on the size of the vessels we examine, do not consider cell-level effects in this work. Although these parameters have been held fixed in the current work, they may change with physiological state or as a result of particular blood pathologies. Evaluating the impact of these on flow is left for a future study. From an analytical perspective, the discretisation parameters also determine the stability and numerical accuracy of LBM simulations. This is done by controlling the computational Mach number of the studied flow (required to be much less than unity) and the single relaxation time used in the HemeLB collision kernel. The number of steps a model is run for combines with $\Delta t$ to determine the physical time observed by the simulation and, where relevant, whether a steady-state has been achieved. Within the LBM generally, and in the implementation of HemeLB used in this study, there are no randomly seeded parameters that would drive aleatoric uncertainty. The pressure boundary conditions applied in HemeLB have been implemented to impose the desired values with a sinusoidal waveform. As a consequence, the available variables are: mean, amplitude, period and phase of the applied profile. With a 3D domain being imposed, velocity boundary conditions can, in principle, be individually tailored to each lattice site within an inlet plane. Within HemeLB this is achieved by applying a weighting to the maximum velocity within the domain. For domains with a circular cross-section the weightings have been chosen to represent a parabolic input profile with a constant maximum velocity. For non-circular cross-sections, the weightings are commonly chosen to represent a parabolic-like profile with the highest weights (and thus highest velocities) applied at the points farthest from a vessel boundary. The applied maximum velocity can be considered as the main input variable for this method. As a consequence, these combine to create a total of $3+4N$ possible variables for simulations with pressure boundary conditions imposed at inlets and outlets and *N* being the total number of such boundaries. When velocity boundary conditions are imposed at the inlets of a domain, this reduces to $3 + I + 4O$ for the desired number of inlets (*I*) and outlets (*O*). Even if the resolution is held constant for a given campaign, it can be seen that the parameter space possible to investigate will grow rapidly in human-scale vasculatures that contain many boundary locations. At the full-human scale, hundreds of boundary locations is not unreasonable. We discuss the range of uncertainties applied to these inputs further in Results when we introduce the individual studies.

When looking at the results of this study for other LBM simulations, the impacts of changing the intrinsic LBM parameters of $\Delta t$, $\Delta x$ and the number of steps will largely be consistent with other codes using the single relaxation time approach. More advanced collision models will demonstrate different characteristics to those observed here, though often at the cost of requiring more tunable parameters. Similarly, there are several choices within the LBM literature for the precise implementation of velocity, pressure and solid boundary conditions and their impact on the flow results may differ significantly to those reported here. However, such observations are not limited to the LBM. All numerical methods have variations of implementation that may impact the quantity of interest being output. The purpose of VVUQ studies is to help understand how this manifests within a given construction.

We also apply the same techniques to an individualised arterial domain that contains a bifurcation as per the canonical examples. This vascular structure contains the radial and ulnar arteries of the left forearm (Fig. 1c). However in this case, we temporally vary the applied maximum inlet velocity, although this temporal profile is kept constant for each case.

We now provide some brief mathematical background for UQ evaluation, for which we cite Wright et al.^29^ in particular. The theoretical framework for evaluating the uncertainty behaviour of a numerical model requires the establishment of a relationship between a set of simulation inputs, $\vec {\xi }$, and the output quantities of interest, *q*. Two commonly deployed frameworks for establishing this relationship are stochastic collocation,

6 $$\begin{aligned} q\left( \vec {\xi } \right) = \sum ^{N}_{j=1} q_j \left( \xi _j \right) L\left( \vec {\xi } \right) , \end{aligned}$$

and the polynomial chaos expansion,

7 $$\begin{aligned} q\left( \vec {\xi } \right) = \sum ^{N}_{j=1} c_j P\left( \vec {\xi } \right) . \end{aligned}$$

Here, the summation representation is developed from *N* evaluations of the numerical model using an appropriately distributed set of $\vec {\xi }$. *L* represents the set of Lagrange polynomials, whilst *P* is a polynomial set chosen to be orthogonal to the input values. In Eq. 7, $c_j$ are coefficients chosen to fit the polynomial representation to the quantities of interest. As per Wright et al.^29^, an approach based on spectral projection has been used to determine these values in this work. The construction of these representations can also enable a method for efficiently computing the output of the numerical model across the input space. In either scheme, for a polynomial of order *p*, a study of a problem with *k* input variables and a full tensor product of the 1D quadrature covering each variable requires $N=(p+1)^k$ model evaluations.

The determination of Sobol indices allows the relative impact of the input variables on the variation of the quantities of interest to be determined. This calculation begins with converting the quantities of interest into a summation of basis functions based on the response to input variables,

8 $$\begin{aligned} q\left( \vec {\xi } \right) = \sum _{u \in F} q_u. \end{aligned}$$

Here, *F* represents the set of possible combinations of input variables. *F* not only includes the single variables contained in $\vec {\xi }$ (first-order Sobol indices), but also the pairwise, and higher order, combinations of them. With $q_0$ representing the mean value of *q*, the $q_u$ terms can be thought of as how the quantity of interest changes in response to varying the selected combination of input parameter(s) defined by *u*. Under this definition, the $q_u$ have zero mean and $D_u$ can then be defined as the variance of $q_u$. The Sobol indices themselves can then be computed as,

9 $$\begin{aligned} S_u = \frac{D_u}{D} = \frac{D_u}{\sum D_u}, \end{aligned}$$

where *D* is the total variance of the quantity of interest. The sum of all possible Sobol indices is one.

For the current work, we have made use of the open-source EasyVVUQ application^29–31^ to conduct the uncertainty study of the HemeLB simulations. This platform is contained within the VECMA^32^ and SEAVEA^33^ toolkits and allows users to define a campaign of simulations that quantify the impact of the chosen range of input parameters on the selected quantities of interest. Importantly, this is achieved without instrumentation of the simulation code itself. Rather, templated input scripts are provided that indicate the simulation input variables along with execution and post-processing instructions. An EasyVVUQ campaign is then constructed that encodes the individual jobs being conducted, executes them, decodes the output and conducts the UQ analysis of the results. The EasyVVUQ application has been developed to interact with a suite of other tools within the VECMA^32^ and SEAVEA^33^ toolkits to enable the efficient execution of VVUQ studies on high-performance computers. This capability is essential to meet the increasing demand for VVUQ to be used to establish the reliability of numerical models. A VVUQ campaign may require multiple hundreds or thousands of individual jobs to fully establish the characteristics of a model. When a single job of a model requires significant compute time and resources by itself, such as for a 3D computational fluid dynamics study, the use of high-performance computers is essential to complete a campaign in a practical amount of time.

## Results

Here we will present a brief description of the studies we conducted in the separate domains described in Methods and some of the most insightful output obtained from these. We will also provide some brief discussion relevant to each case. In these studies, we will be applying a velocity boundary condition to the inlets and pressure boundary conditions to the outlets. This has been chosen to represent the ‘typical’ configuration used for vascular simulations. In all cases discussed here, domains are initialised with a constant pressure of 0mmHg. There are no aleatoric components within these simulations.

In this study we have used two measures to evaluate the relative influence of input parameters in the uncertainty of the quantities of interest. Firstly we display the observed distribution of the quantity of interest around the mean value. Context will be provided to this by the bound of a standard deviation around the mean and bounds indicating the 1% and 99% distributions of observed results. Secondly, we present the first-order Sobol indices calculated over the spatial or temporal domain of interest. The change in these quantities will indicate the relative influence of the particular input parameters on the variation of the quantity of interest at that point. In these plots, the ‘Index’ along the x-axis represents the count of grid sites along the central axis of the domain.

### Canonical bifurcation

A set of campaigns was conducted to investigate the influence of algorithmic and boundary condition parameters on flow within the canonical bifurcation domain (Fig. 1a). This domain consists of three equal length arms, with two running with axes aligned and the third separating off at an angle of 45^∘^ to the downstream direction of flow. In all cases, studies were conducted at two levels of domain resolution corresponding to domain radii of approximately 20 and 40 $\Delta x$. All sections of the domain had constant radius. The arms of the bifurcation were each five times longer than the radius. Due to this, the main channel (combining the inlet branch and the outlet path following the straight path from the inlet) was 10 radii in total length (200 or 400 $\Delta x$). The side branch splits from the central point of the main channel and is 5 radii in length from this point. For each domain, several studies were undertaken. We have intentionally separated our studies to examine the respective effects of algorithmic and boundary parameters on our simulations.

In the first study, the LBM algorithmic parameters of $\Delta x$, $\Delta t$ and the number of simulation steps are uniformly varied between selected bounds. Between the two resolutions, the ranges of these respective unknowns were set to ensure a consistent range of physical values between them. In the coarser case, the ranges chosen were $\Delta x \in \left[ 5 \times 10^{-5}, 1.5 \times 10^{-4} \right]$ m, $\Delta t \in \left[ 6 \times 10^{-5}, 1.2 \times 10^{-4} \right]$ s and number of steps $\in \left[ 2500, 5000 \right]$. The boundary parameters were chosen to be a constant inlet velocity of 0.001 m/s, and outlet parameters to provide a constant pressure condition. These values have been chosen to ensure stable simulations occur across all selections in a campaign and corresponds to Reynolds number values below 1.5. To maintain consistency in the finer case, the $\Delta x$ values were halved, $\Delta t$ values quartered and the number of steps scaled by four.

The remaining cases examined combinations of the outlet parameters. Here two cases examined the inlet velocity combined with the variation of the mean, amplitude, phase and period at the respective outlets. The third case held the inlet velocity constant and varied the boundary parameters over both outlets. The final case combined all significant boundary parameters.

For these boundary condition cases, the maximum velocity provided to the inlet and mean, amplitude, phase and period of the sinusoidal outlet profile were varied. As these values were set based on physical values, the same parameter ranges were used for both resolutions. Here they were chosen to be: inlet velocity $\in \left[ 0.0, 0.005 \right]$ m/s, outlet mean $\in \left[ 0.0, 0.00025 \right]$ mmHg, outlet amplitude $\in \left[ 0.0, 0.00025 \right]$ mmHg, outlet period $\in \left[ 1.0, 2.0 \right]$ s and outlet phase $\in \left[ 0.0, 0.05 \right]$ rad. These values were chosen to facilitate stable simulations with a uniform distribution being imposed in these ranges. The respective algorithmic parameters were chosen to be $\Delta x = 5 \times 10^{-5}$ m, $\Delta t = 6 \times 10^{-5}$ s and 5000 steps for the coarser domain and $\Delta x = 2.5 \times 10^{-5}$ m, $\Delta t = 1.5 \times 10^{-5}$ s and 20000 steps for the finer domain. The largest Reynolds number observed in these tests was approximately 2.5.

**Figure 2:**
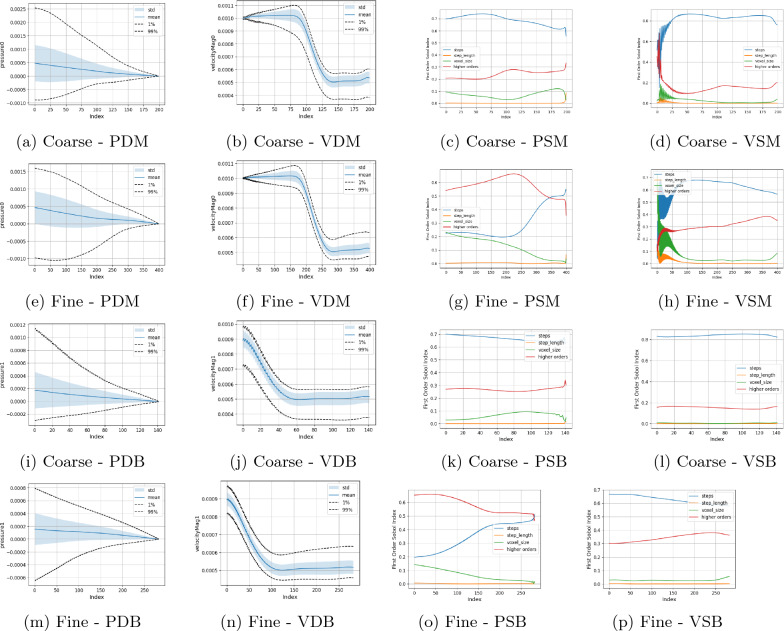
Uncertainty and parameter sensitivity analysis of the pressure and velocity within the coarse and fine bifurcation geometries determined with a third-order polynomial chaos expansion. Algorithmic parameters of the LBM have been varied here with constant boundary conditions. Larger images are provided in the Supplementary Information. The identifying abbreviations are: V = velocity, P = pressure; D = distribution, S = Sobol indices; M = main channel, B = side branch.

In all campaigns, a third-order polynomial chaos expansion was used to evaluate the uncertainty. With the exception of Fig. 2 (discussed further below), comparison of Sobol index results with higher order expansions illustrated no change in the patterns observed, indicating that this choice of analysis order and number of trials within a campaign was justified. As HemeLB requires the domain to be pre-processed to the desired resolution, rather than the discretised domain being generated based on an input parameter provided to the simulation, the mechanism for constructing an EasyVVUQ campaign meant that the spatial discretisation could not be varied within a single UQ campaign for this study. The effect of spatial resolution, however, can be observed through comparison of the two grid sizes studied here.

Two fundamental quantities of interest are investigated, namely the pressure and velocity along the central axes of the domain branches. The measurement domains for the main channel (red) and side branch (blue) are also indicated in Fig. 1a. Examining these parameters allows this study to observe how the fundamental macroscopic quantities of the flow vary spatially across the domain in response to input parameter change. Here, due to the circular cross-section of the canonical bifurcation, we use the central axis as a representative measure of flow throughout the branch. In all plots, the ‘Index’ along the x-axis represents the count of grid sites along the central axis of the main channel or side branch. For the boundary condition studies, in (a), (b), (e) and (f) of the respective Figures, we present the distribution of the macroscopic quantities of interest with the mean, standard deviation and 1% and 99% confidence intervals being indicated. In (c), (d), (g) and (h) we indicate the first-order Sobol indices for the input variables. The ‘higher orders’ plots indicate the variation resulting from simultaneous variation of the input parameters from their default values. In the results presented here, $\Delta x$ = $\texttt {voxel\_size}$, $\Delta t$ = $\texttt {step\_length}$, number of steps = $\texttt {steps}$. For cases examining boundary condition variations, the $\texttt {inlet\_max}$ is the input velocity, $\texttt {outlet0\_mean}$, $\texttt {outlet0\_amplitude}$, $\texttt {outlet0\_phase}$ and $\texttt {outlet0\_period}$ are the mean, amplitude, phase and period of the sinusoidal outlet pressure condition. All plots have been oriented such that the flow direction is left-to-right.

#### Changing algorithmic parameters

**Figure 3:**
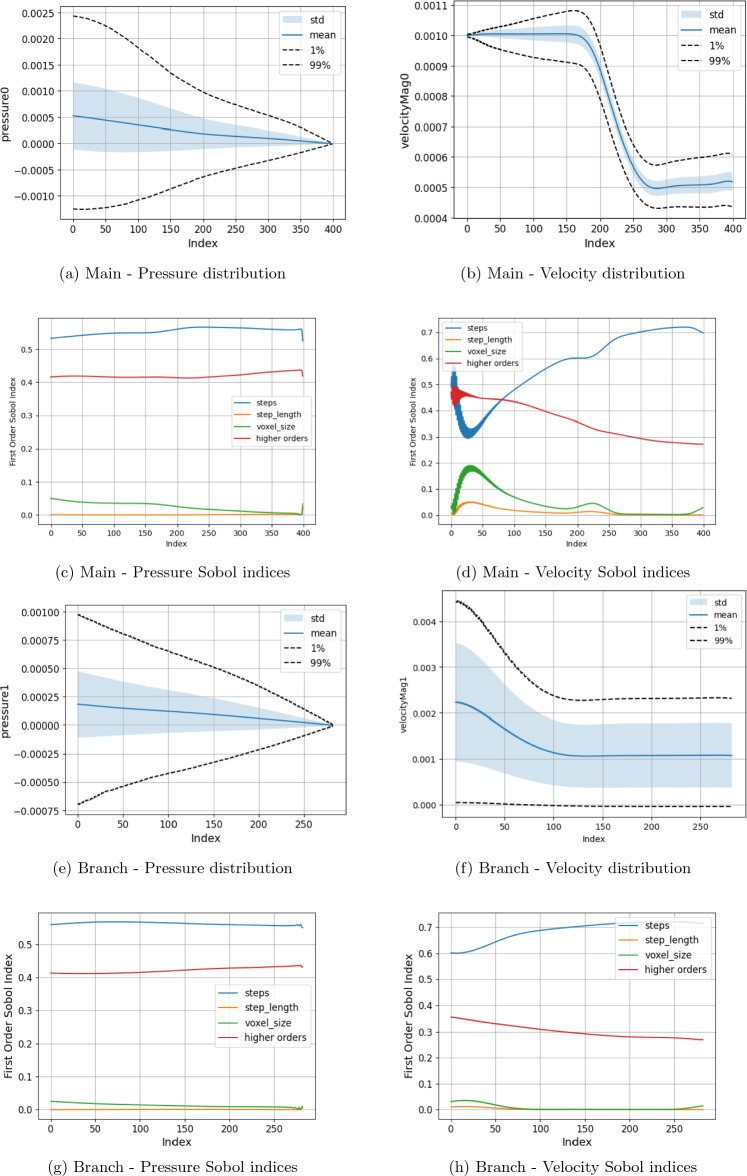
Uncertainty and parameter sensitivity analysis of the pressure and velocity within the bifurcation geometry determined with a ninth-order polynomial chaos expansion. Algorithmic parameters of the LBM have been varied here with constant boundary conditions.

Figure 2 illustrates the statistical analysis in the main channel and side branch, respectively, of the canonical bifurcation. The number of simulation steps is the most influential individual parameter, generally followed by the grid spacing. This strongly indicates the impact of temporal solution convergence on the results of numerical simulations. However, as the mean values of the quantities of interest observed in the coarse and fine resolution studies are very close, it can be inferred that the spatial convergence has been achieved by the chosen domains. The lack of temporal convergence in the results is also manifest in the Sobol indices reported by the analysis. Even on increasing the order of the polynomial chaos expansion up to ninth order we found that the Sobol indices did not converge to a consistent set of values for the higher resolution model. Despite this, at all tested orders, a general trend was observed where the number of simulation steps was the most influential single parameter, representing around 40–60% of variation for both the pressure and velocity outputs. The shape of the average value of the observed quantities but the outer 1%/99% confidence intervals varied slightly with analysis order. The results of a ninth-order analysis are presented in Fig. 3. Further results are provided in the Supplementary Information. The combination of these results demonstrates the importance of converged simulations for a reliable UQ analysis.

**Figure 4:**
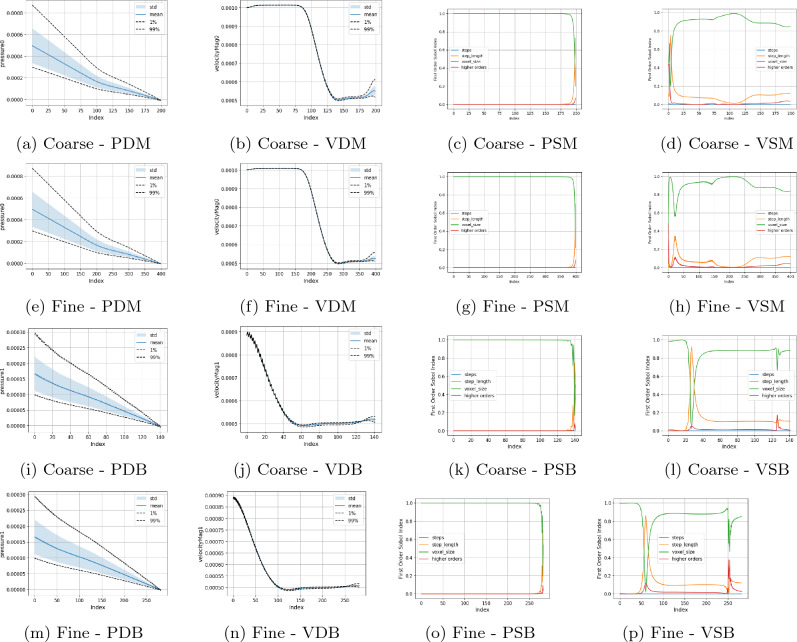
Converged uncertainty and parameter sensitivity analysis of the pressure and velocity within the coarse and fine bifurcation geometries determined with a third-order polynomial chaos expansion. Algorithmic parameters of the LBM have been varied here with constant boundary conditions. Larger images are provided in the Supplementary Information.

To remove the effect of temporal convergence, we repeated the algorithmic studies with the number of steps being simulated increased by a factor of ten. The effect of this can be seen in the Sobol index plots of Fig. 4 (which now remain constant if the order of the polynomial chaos expansion is increased) where the steps parameter has no impact on either the pressure or velocity quantities of interest examined. This now isolates the variation within the domain to those caused by the changing discretisation parameters, to which the voxel size was the major contributor. In the shorter simulations, the mean values of the coarse and fine resolution studies were very similar suggesting that grid independence had been achieved at this point and the variation of results were due to the quantities of the input values examined. This has been verified with the removal of the influence of the time steps on the simulation results with Fig. 4 showing nearly identical results for the distributions of pressure and velocity. In these findings, the only significant variation of the results is seen in the velocity at the outlets of the domain. A more detailed analysis of the grid convergence behaviour and error estimation of HemeLB with these boundary conditions is provided in Nash et al.^19^.

#### Changing boundary parameters

**Figure 5:**
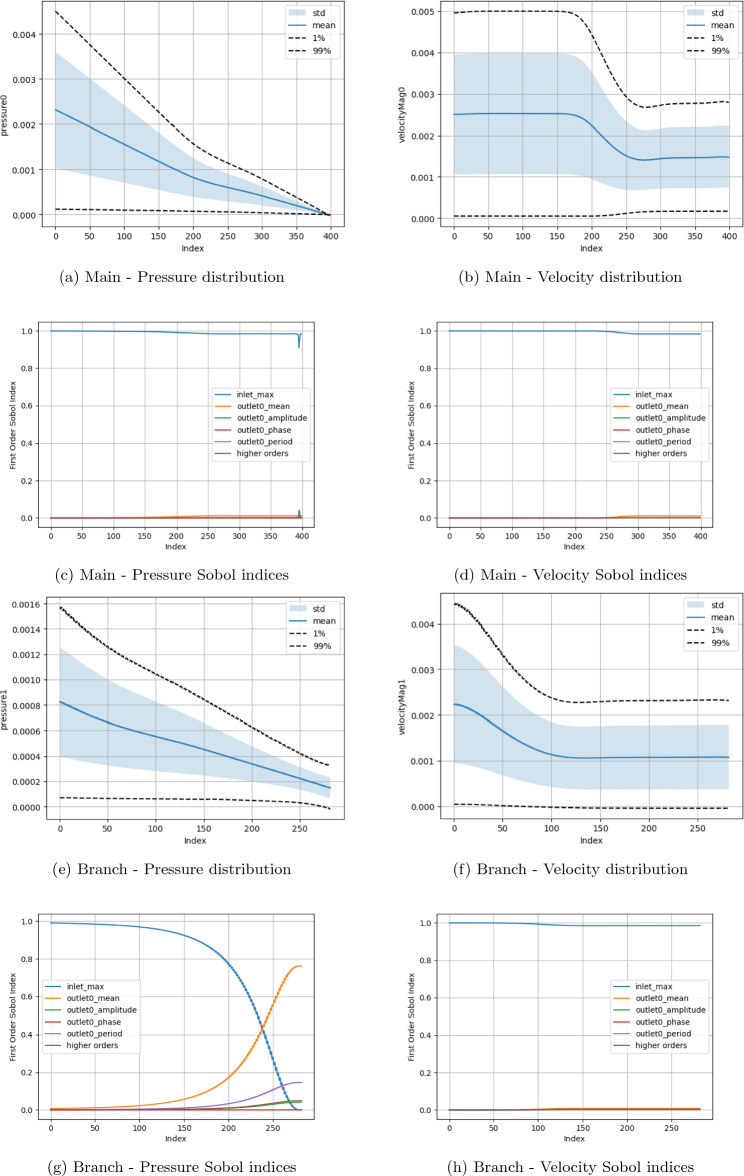
Uncertainty and parameter sensitivity analysis of the pressure and velocity within the fine bifurcation geometries determined with a third-order polynomial chaos expansion. Boundary condition BC1.

**Figure 6:**
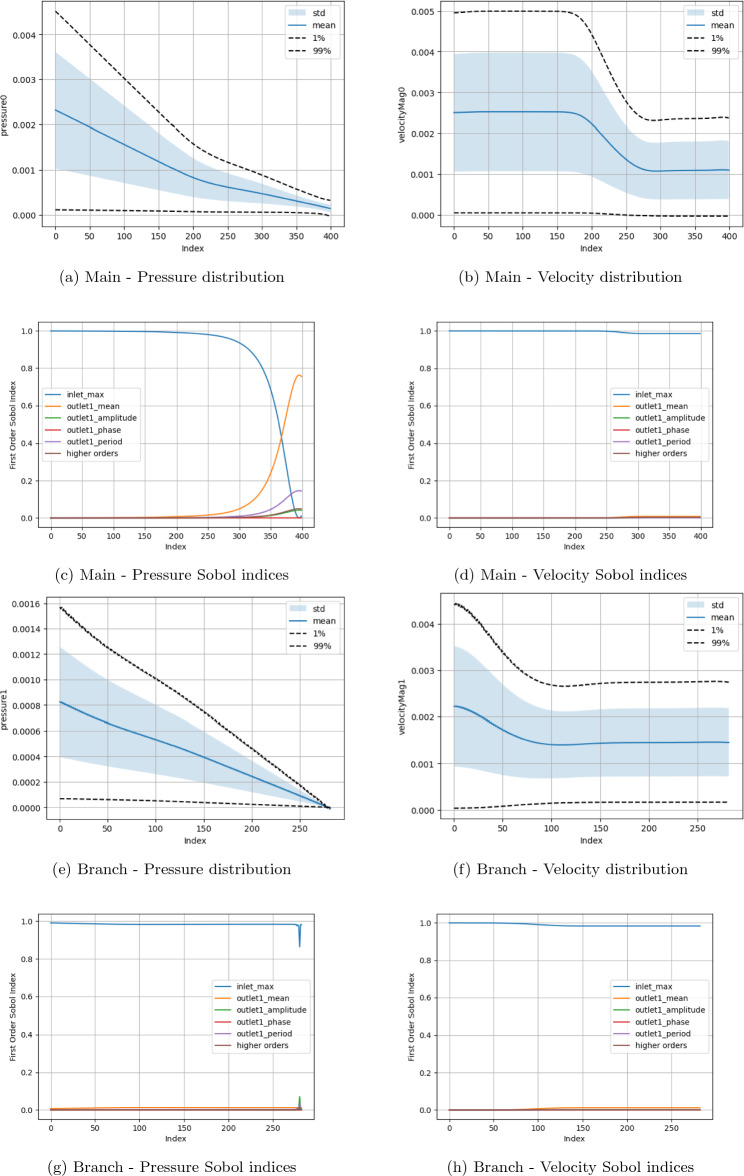
Uncertainty and parameter sensitivity analysis of the pressure and velocity within the fine bifurcation geometries determined with a third-order polynomial chaos expansion. Boundary condition BC2.

**Figure 7:**
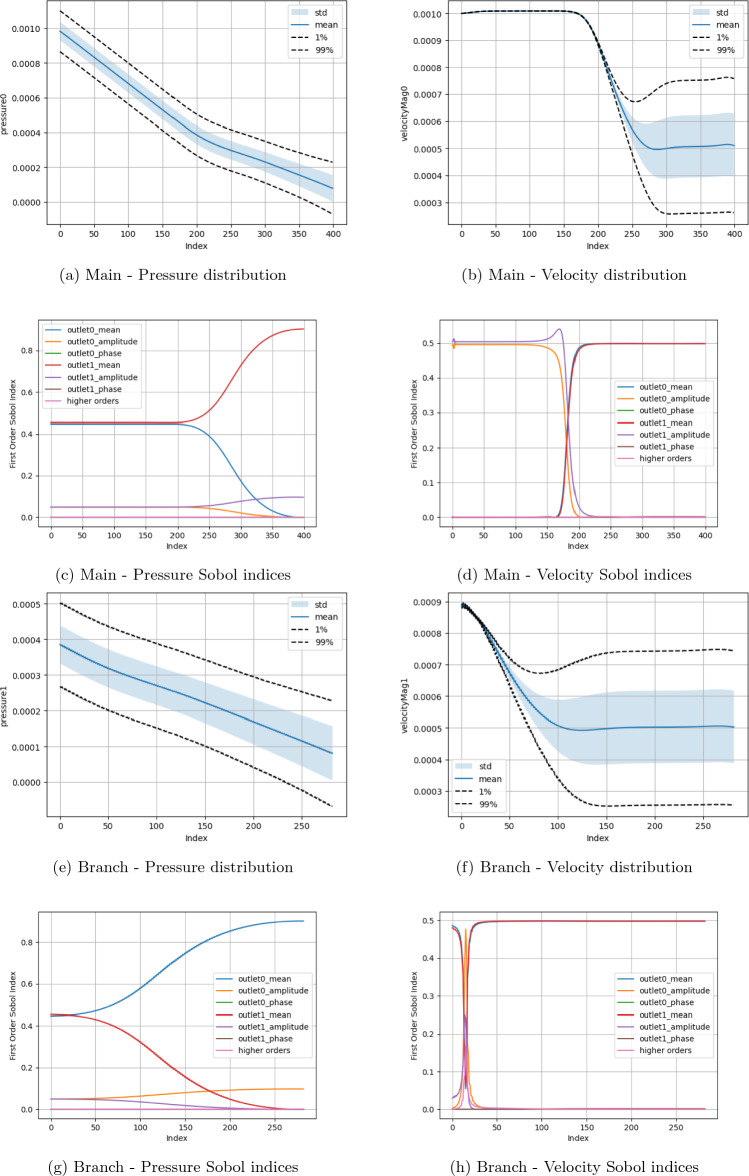
Uncertainty and parameter sensitivity analysis of the pressure and velocity within the fine bifurcation geometries determined with a third-order polynomial chaos expansion. Boundary condition BC3.

**Figure 8:**
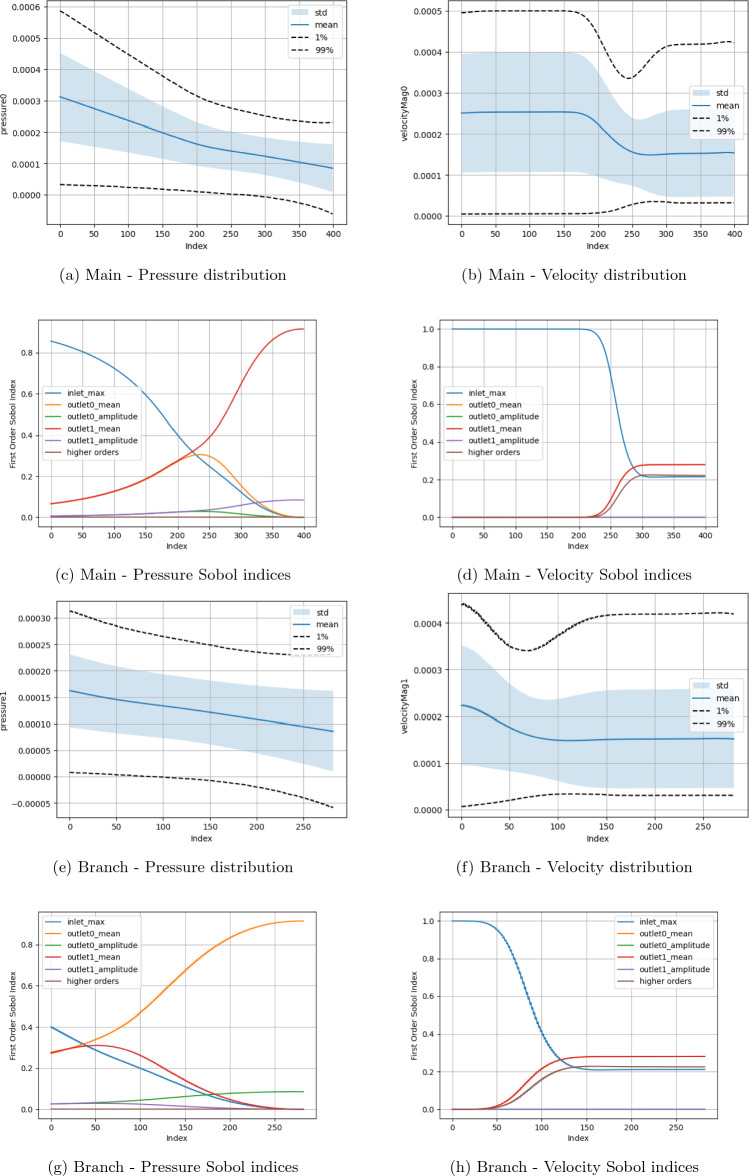
Uncertainty and parameter sensitivity analysis of the pressure and velocity within the fine bifurcation geometries determined with a third-order polynomial chaos expansion. Boundary condition BC4.

In Figs. 5, 6, 7, and 8 we present the Sobol index and distribution results in the campaigns varying the boundary condition parameters. Figure 5 varies the inlet and all branch outlet components (BC1) and Fig. 6 the inlet and all main channel outlet components (BC2). In Fig. 7 (BC3) both pressure outlets are varied though with the period being held constant as it would be in a vascular domain. In Fig. 8 (BC4), the inlet is varied along with the mean and amplitude of both pressure boundaries are varied, the period and phase of the pressure outlets are set to their default values as the previous tests showed these to be less influential. As a representative case, the Sobol index results in Fig. 8 remained constant if the order of the polynomial chaos expansion was increased. The coarse results were observed to be very similar to the fine results under boundary condition variation. These are provided in the Supplementary Information section. Within the Supplementary Information, we also provide the results from a campaign where we increased the maximum value applied for the mean and amplitude of the pressure condition by a factor of 100. In these cases, it can be seen that both the computed values and the parameters influencing these in different regions are very different to those presented here. This is despite the headline results for LBM Mach number indicating a stable simulation.

In Figs. 5 and 6, where only one of the pressure outlets is being varied, it can be seen that the chosen value of the inlet velocity is the dominating factor in the variation of both the pressure and velocity quantities of interest. This controls nearly 100% of the velocity variation throughout both the main channel and the side branch. Following the bifurcation, the section of the domain with the constant pressure condition on the outlet also sees the inlet velocity dominating the variation of pressure within this region. The area of exception is the pressure in the region beyond the bifurcation leading to the variable outlet. Here, the mean pressure applied at the outlet becomes the dominating factor by describing some 75% of pressure variation in the proximity of the outlet. A further 15% of the total variation is then described by the period of the oscillating outlet. The amplitude of the oscillation appears to have a minimal impact in this setting. This is in contrast to the higher pressure campaign where the amplitude of the pressure condition had a much more significant impact on the velocity profiles observed in the domain. The distribution plots also indicate how the variation of the outlet condition changes the distribution of flow within the domain. In both Figs. 5 and 6, the outlet with the variable pressure has an outlet velocity some 50% greater than that seen in the region adjacent to the constant pressure outlet based on the values used in this campaign. This illustrates that the outlet boundary condition is controlling flow rather than the geometry of the domain.

When only the pressure outlets are being changed (Fig. 7), a high degree of symmetry between the analysed parameters is recorded. This would appear to again indicate that for the chosen geometric configuration, the asymmetry of the domain again does not impose a significant impact. This is potentially related to the flow distance between the point of observation and the pressure outlets being the same in this case, leading to similar pressure gradients being imposed. Before the bifurcation, the small variation of velocity within the channel is dominated by the amplitude of the oscillating boundary conditions. Beyond the bifurcation, the combined effects of the chosen mean value of the pressure boundary conditions describes approximately 100% of the changes in velocity. The pressure variable is almost fully accounted for by the mean pressure value of the local outlet with minor contribution from that outlet’s amplitude value. Beyond the bifurcation, the range of the velocity distribution if relatively narrower than that seen when only one outlet was varied.

The combined boundary case (Fig. 8) also records a combination of the observed statistical results. Before the bifurcation, the inlet velocity is solely responsible for the velocity variation in the domain and well over 50% of the pressure variance. The mean pressure of the outlet boundaries become increasingly more influential on the pressure distribution as flow progresses towards the respective outlets. Beyond the bifurcation they are also contribute significantly to the variation in velocity. Indeed, in this region the mean pressure values of the outlets respectively contributes some 30% of the velocity variation compared to the inlet velocity, and combined higher order effects, only providing just over 20%. The amplitude of the pressure boundary condition makes no contribution to the velocity variation.

### Personalised arteries

**Figure 9:**
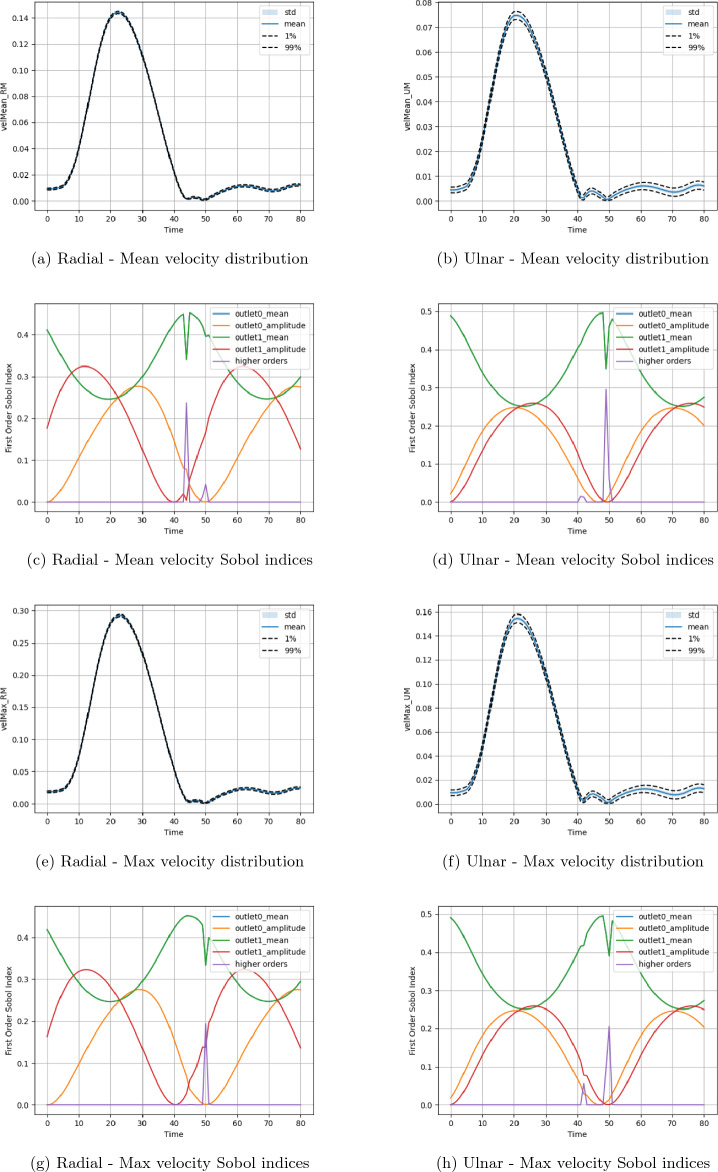
Uncertainty and parameter sensitivity results for the mean and maximum velocities at the analysis plane in the radial and ulnar arteries of the personalised vessels. In all plots, the ‘Time’ along the x-axis represents the time series taken through the heartbeat section of the simulation.

The personalised forearm vessels presented in Fig. 1 were discretised to a resolution of $\Delta x = 5\times 10^{-5}$ m. A time step of $\Delta t = 5\times 10^{-6}$ s was applied for a total of 325000 steps—simulating a physical time of 1.625 s. The first 0.8 s of this time was dedicated to an initialisation period of flow within the domain. For the second 0.8 s a heartbeat profile was applied to the inlet of the domain via a velocity inlet condition and a parabolic-like spatial profile. The initial state of the flow was stationary. These are illustrated in Fig. 1d and e respectively. In our previous work^34^ we observed that for this domain and inlet condition, a single heartbeat cycle was sufficient to achieve periodic flow in the domain. At the outlets of the vessels, a sinusoidal pressure profile was applied as for the canonical bifurcation. The input parameters for this uncertainty study were the mean and amplitude of these profiles. We decided to apply a range of between 0 mmHg and 0.2 mmHg for each of these values. This gave a total of four input parameters and, by using a third-order polynomial chaos expansion to orchestrate the campaign, required 256 individual simulations to be completed. The quantities of interest that we chose to examine in this case were the mean and maximum velocities at plane located midway along the radial and ulnar arteries (upper and lower vessels, respectively, in Fig. 1c). We extracted these values at multiple time points during the heartbeat profile section of the simulation. Due to the organic shape of vascular structures, defining central lines for data output as done for the canonical bifurcations is not straightforward with current features of HemeLB. Instead, we have chosen to study these quantities of interest as they are more representative of the manner in which a general CFD or medical imaging study would analyse a patient’s vessels. The distribution and Sobol indices of the uncertainty study are presented in Fig. 9. Under these conditions, there is generally a larger uncertainty in the flow in the ulnar artery compared to the radial artery. This is particularly manifest in the diastolic region of low flow rates in the second half of the profile. We believe that this is due to the geometry of the bifurcation in the personalised vessels making the ulnar artery more susceptible to flow variation. It can be estimated however that the magnitude of mean velocity variation is similar in both vessels. In the plots of Sobol indices, the mean values applied at the radial ($\texttt {outlet1\_mean}$) and ulnar ($\texttt {outlet0\_mean}$) arteries are equally influential determining the quantities of interest. The relative influences of the radial ($\texttt {outlet1\_amplitude}$) and ulnar ($\texttt {outlet0\_amplitude}$) amplitudes vary throughout the time period and are slightly more influential to the radial artery values compared to the ulnar artery. In the Supplementary Information, we illustrate the normal distribution of the mean and maximum velocities observed in the personalised vessels. This data has been sampled based on the polynomial chaos expansion of the observed simulation data.

## Discussion

Within the LBM, it is known that the choice of discretisation parameters can have a significant impact on the stability and accuracy of a simulation^25^. The studies we have conducted here provide a numerical illustration of this impact and how it manifests across the key macroscopic variables of pressure and velocity. Regarding the parameters of the LBM, the outcomes we observed can be seen to be consistent with existing knowledge of the method. Being an explicit method, the number of steps of a simulation will be critical in a simulation of transient phenomena. Generally speaking however, the choice of $\Delta x$ has a greater influence on our observed quantities of interest than $\Delta t$. The conversion of these parameters into the LBM (particularly the single relaxation time model used in this study) occurs in the calculation of the relaxation time ($\tau$) used in collision. The conversion of kinematic fluid viscosity ($\nu$) from physical to dimensionless simulation (lattice) units ties these together through Eq. 5. This conversion indicates that the grid spacing has a greater impact on the value of $\tau$ in a simulation due to the power of two and as a result the rate at which the fundamental LBM mesoscopic variables are updated at each time step.

In the studies presented here, the impact of domain resolution needed to be assessed through independent campaigns rather than being integrated within a single study. This was due to the HemeLB code requiring the pre-processing of simulation geometries to create domains rather that this being integrated within the simulation process. This process was not sufficiently automated to permit its inclusion within this study. However, a particular examination of resolution impact on flow would be an interesting and important avenue of further study.

The outlet boundary conditions imposed within HemeLB here assume that flow is fully developed with no secondary flows at these locations. For the canonical bifurcations, with the low Reynolds number flows being studied, this is a reasonable assumption to make for the straight branches being utilised in these studies. For the personalised vessels, the laminar regime Reynolds number, significant distance from the bifurcation to the outlets planes and the generally straight and constant cross-section of the arteries are also sufficient to indicate that this boundary assumption is valid in this case.

As has been conducted in previous analyses with EasyVVUQ^10^, we can assess how the model magnifies or attenuates the variation of the input parameters into the quantities of interest. The ratio of these coefficients of variations (CVR) is given by Edeling et al.^10^ as,

10 $$\begin{aligned} CVR = CV\left( \vec {q} \right) / CV\left( \vec {\xi } \right) = \left( \frac{1}{M} \sum _{m=1}^{M} \frac{\sigma _{q_m}}{\mu _{q_m}} \right) / \left( \frac{1}{D} \sum _{d=1}^{D} \frac{\sigma _{\xi _d}}{\mu _{\xi _d}} \right) , \end{aligned}$$

where the study has *M* quantities of interest (*q*) and *D* input parameters ($\xi$). The mean, $\mu$, and standard deviation, $\sigma$ of $\xi$ are known from the imposed distributions and can be calculated from the observed distributions for *q*. In our assessment, we have taken *M* to represent all quantities of interest in a particular campaign, spatially or temporally averaged as appropriate. In Table 1 we present the coefficients of variation observed in each study in this work based on a third-order polynomial chaos expansion. Coefficients of variation for the higher pressure boundary condition cases are provided in the Supplementary Information. Whilst none of our campaigns involve intentional aleatoric variation, in that we have no randomly seeded parameters within our algorithms, the CVR can be seen as a way of assessing the relationship between deterministic and stochastic elements within the construction of the models used in our campaigns. A CVR not equal to one indicates the presence of model non-linearities that may manifest in stochastic behaviour in certain circumstances even without random seeds.

**Table 1:** Coefficients of variation of input parameters and quantities of interest as generated by the LBM implemented within HemeLB using a third-order polynomial chaos expansion.

Study	$CV\left( \vec {q} \right)$	$CV\left( \vec {\xi } \right)$	*CVR*
Coarse Bifurcation Algorithmic	0.8329	0.2245	3.7097
Coarse Bifurcation Algorithmic Converged	0.2033	0.2245	0.9056
Coarse Bifurcation BC1	0.6586	0.5004	1.3163
Coarse Bifurcation BC2	0.6419	0.5004	1.2829
Coarse Bifurcation BC3	0.2510	0.5774	0.4347
Coarse Bifurcation BC4	0.6693	0.5774	1.1592
Fine Bifurcation Algorithmic	0.8573	0.2245	3.8183
Fine Bifurcation Algorithmic Converged	0.1315	0.2245	0.5859
Fine Bifurcation BC1	0.6582	0.5004	1.3154
Fine Bifurcation BC2	0.6432	0.5004	1.2854
Fine Bifurcation BC3	0.2455	0.5774	0.4252
Fine Bifurcation BC4	0.6944	0.5774	1.2023
Forearm Arteries	0.0731	0.5774	0.1268

From these results, it can be observed that the LBM will generally amplify the uncertainty present within the input parameters. This appears to be particularly important when transient effects still being present within the analysis due to the particular set of parameters chosen for the campaign. Within the algorithmic studies, the CVR is significantly reduced when the temporal component of results is removed by the converged results. As for the Sobol indices for the canonical bifurcation, the CVR values also varied with the order of the polynomial chaos expansion. However, when the general trend of the Sobol index behaviour was established (at and above sixth-order analysis), the amplification of input uncertainty remained. For the bifurcation case study, the variation of boundary conditions in all combinations, typically saw the coefficient of variation of the quantities of interest being 15–30% larger than that of the input parameters. That the LBM is more robust when a fixed set of algorithmic parameters is chosen is logical given the relationship to the physics being solved, the discretisation parameters used and the fixed physical time being simulated. The exception among the bifurcation cases was the boundary condition campaign where only the pressure outlets were varied, here the variation is damped. In the case of the forearm arteries, where again only pressure conditions were being altered, the input variation was also significantly damped. Whilst the arteries case did analyse the flow results in quite a different way to the bifurcation, with these campaigns each maintaining a constant applied velocity this indicates that this parameter may be much more influential on whether variations are damped or magnified and indicates that care should be taken when this parameter is being changed. On its own, small pressure variations seem to have less of an impact. When larger pressure values are applied, the bifurcation case BC3 amplifies the variation of the input variables to a similar order of magnitude to the other boundary condition cases. Domain resolution does not appear to have a significant impact on the coefficient of variation computed for a campaign.

We observed in the canonical bifurcation that the asymmetry of the geometry did not lead to an asymmetry in the influence of variables on the quantities of interest as judged by the first-order Sobol indices. We believe that in this case although the geometry is asymmetric the distance along the flow channel to the pressure boundaries was the same for either branch leading to equal pressure gradients in either the main channel or branch direction. Examining the impact of bifurcation branch length on the quantities of interest would likewise be another avenue of investigation that would be particularly relevant to studies of vascular blood flow given the proliferation of such cases throughout the arterial and venous networks. Deductions can be made regarding this as pressure gradients can be estimated based on the imposed pressure and known branch length. As ever, careful choice and imposition of data at outlet branches is shown to be crucial.

As further example of the use of UQ techniques used with the LBM in high-performance computing in the wider scientific literature we can cite the recent work of Falcucci et al.^35,36^. This work uses the LBM to study the complex flow structures generated around the skeletal structure of deap-sea sponges. In this study, the bounds of the observed range for flow data such as helicity, enstrophy and drag coefficients are provided to give further context to the variability of their simulations.

As can be noted from the studies implemented in this paper, uncertainty trends can be very problem dependent. Determination of both input parameters and evaluation of quantities of interest can lead to different outcomes that may influence the potential use of a numerical model in practice. These challenges only serve to reinforce the need to ensure that appropriate VVUQ is applied to numerical models used to make day-to-day decisions.

## Conclusion

In this paper we have made use of the open-source tool EasyVVUQ to conduct uncertainty studies on the LBM as deployed in the HemeLB code. Whilst focussed on assessing aspects of the LBM itself, we have chosen to study a flow domain that is representative of the geometric characteristics observed in vascular domains. Particular focus has been given to bifurcations where we study an idealised geometry with a variety of combinations of input parameters imposed to reflect their use in vascular simulations. An initial campaign varied algorithmic and discretisation parameters of the LBM to evaluate how the method itself may contribute to variation in key quantities of interest–pressure and flow velocity—throughout the domain. Following transient effects related to achieving steady-state, the grid spacing of the simulations was consistently observed to be more influential in the output uncertainty than the size of the time step used. The impact of parameters in velocity and pressure boundary conditions was investigated in a set of campaigns that illustrated how the choice of inlet velocity, in particular, can dominate the computation of observed pressure and velocity throughout the domain. When asymmetry was introduced to the outlet pressure boundary conditions, the bifurcation provided a transitional location for their local effects. We conclude with an uncertainty study in a personalised set of arteries from the left forearm. This study provided insight into how the propagation of a transient inlet velocity profile is modified by varying outlet pressure conditions. In both vessels, the mean and maximum velocities were maintained within relatively tight bounds for the range of boundary parameters tested. The chosen mean values of the outlets generally had the greatest effect. The geometry of the personalised vessels also manifested in a different distribution of Sobol indices for the varied parameters between the radial and ulnar vessels. A coefficient of variation analysis conducted on each campaign evaluated how the magnitude of the input parameter change was manifested in the the variability of the output quantities. The algorithmic variation campaigns saw significantly greater magnification of the error than those campaigns only modifying boundary conditions. It can also be noted that the boundary condition campaigns that did not involve modification of the inlet velocity condition (BC4 and the personalised vessels) actually observed a suppression of variability. This result particularly highlights the care that must be taken when velocity boundary conditions are required. The variety of results in our various studies highlights how problem sensitive conducting uncertainty evaluations can be. The choice of boundary conditions can be seen to exert a significant impact on the uncertainty characteristics of the flow simulations. However the magnitude of these impacts may not necessarily be obvious ahead of conducting an explicit UQ study.

Uncertainty quantification is rapidly becoming a crucial component of the transition of numerical tools from the research domain towards deployment in wider contexts. This paper has illustrated how open-source tools can be utilised to assess the uncertainty characteristics of computational fluid dynamics codes under a range of numerical and boundary conditions. With a particular insight towards blood flow simulations, this work has the potential to help guide numerical decision making for future research in this setting.

### Supplementary Information

Supplementary Information.

## Data Availability

The datasets used and/or analysed during the current study available from the corresponding author on reasonable request.

## References

[1] Viceconti M, Pappalardo F, Rodriguez B, Horner M, Bischoff J, Musuamba-Tshinanu F (2021). In silico trials: Verification, validation and uncertainty quantification of predictive models used in the regulatory evaluation of biomedical products. Methods.

[2] Software as a medical device (samd), https://www.fda.gov/medical-devices/digital-health-center-excellence/software-medical-device-samd. (2018).

[3] Medical devices: software applications (apps). https://www.gov.uk/government/publications/medical-devices-software-applications-apps. (2022).

[4] Assessing credibility of computational modeling through verification and validation: Application to medical devices. https://www.asme.org/codes-standards/find-codes-standards/v-v-40-assessing-credibility-computational-modeling-verification-validation-application-medical-devices. (2018).

[5] Wan S, Sinclair RC, Coveney PV (2021). Uncertainty quantification in classical molecular dynamics. Philos. Trans. R. Soc. A: Math. Phys. Eng. Sci..

[6] Vassaux M, Wan S, Edeling W, Coveney PV (2021). Ensembles are required to handle aleatoric and parametric uncertainty in molecular dynamics simulation. J. Chem. Theory Comput..

[7] Coveney PV, Groen D, Hoekstra AG (2021). Reliability and reproducibility in computational science: implementing validation, verification and uncertainty quantification in silico. Philos. Trans. R. Soc. A: Math. Phys. Eng. Sci..

[8] Tripathy RK, Bilionis I (2018). Deep UQ: Learning deep neural network surrogate models for high dimensional uncertainty quantification. J. Comput. Phys..

[9] Chen P, Quarteroni A (2015). A new algorithm for high-dimensional uncertainty quantification based on dimension-adaptive sparse grid approximation and reduced basis methods. J. Comput. Phys..

[10] Edeling W, Arabnejad H, Sinclair R, Suleimenova D, Gopalakrishnan K, Bosak B, Groen D, Mahmood I, Crommelin D, Coveney PV (2021). The impact of uncertainty on predictions of the CovidSim epidemiological code. Nat. Comput. Sci..

[11] Constantine PG, Dow E, Wang Q (2014). Active subspace methods in theory and practice: Applications to kriging surfaces. SIAM J. Sci. Comput..

[12] Edeling W (2023). On the deep active-subspace method. SIAM/ASA J. Uncertain. Quantif..

[13] Fukumizu K, Leng C (2014). Gradient-based kernel dimension reduction for regression. J. Am. Stat. Assoc..

[14] Edeling, W., Vassaux, M., Yang, Y., Wan, S., Guillas, S. & Coveney, P. V. Global ranking of the sensitivity of interaction potential contributions within classical molecular dynamics force fields. Preprint at Research Square. https://doi.org/10.21203/rs.3.rs-3379397/v1, (2023).

[15] Heme, L.B. www.hemelb.org. (2019).

[16] Mazzeo MD, Coveney PV (2008). HemeLB: A high performance parallel lattice-Boltzmann code for large scale fluid flow in complex geometries. Comput. Phys. Commun..

[17] Bernabeu MO, Nash RW, Groen D, Carver HB, Hetherington J, Krüger T, Coveney PV (2013). Impact of blood rheology on wall shear stress in a model of the middle cerebral artery. Interface Focus.

[18] Bernabeu MO, Jones ML, Nielsen JH, Krüger T, Nash RW, Groen D, Schmieschek S, Hetherington J, Gerhardt H, Franco CA, Coveney PV (2014). Computer simulations reveal complex distribution of haemodynamic forces in a mouse retina model of angiogenesis. J. R. Soc. Interface.

[19] Nash RW, Carver HB, Bernabeu MO, Hetherington J, Groen DK, Krüger T, Coveney PV (2014). Choice of boundary condition for lattice-Boltzmann simulation of moderate-Reynolds-number flow in complex domains. Phys. Rev. E.

[20] Groen D, Richardson RA, Coy R, Schiller UD, Chandrashekar H, Robertson F, Coveney PV (2018). Validation of patient-specific cerebral blood flow simulation using transcranial doppler measurements. Front. Physiol..

[21] McCullough JWS, Richardson RA, Patronis A, Halver R, Marshall R, Ruefenacht M, Wylie BJN, Odaker T, Wiedemann M, Lloyd B, Neufeld E, Sutmann G, Skjellum A, Kranzlmueller D, Coveney PV (2021). Towards blood flow in the virtual human: Efficient self-coupling of hemelb. Interface Focus.

[22] Succi S (2001). The Lattice Boltzmann Equation for Fluid Dynamics and Beyond.

[23] Mohamad AA (2011). Lattice Boltzmann Method: Fundamentals and Engineering Applications with Computer Codes.

[24] Guo, Z. & Shu, C. Lattice Boltzmann method and its applications in engineering. *WORLD SCIENTIFIC* (2013). 10.1142/8806. https://www.worldscientific.com/doi/abs/10.1142/8806

[25] Krüger T, Kusumaatmaja H, Kuzmin A, Shardt O, Silva G, Viggen EM (2017). The Lattice Boltzmann Method: Principles and Practice.

[26] Succi S (2018). The Lattice Boltzmann Equation: For Complex States of Flowing Matter.

[27] Ladd AJC (1994). Numerical simulations of particulate suspensions via a discretized Boltzmann equation. Part 1. Theoretical foundation. J. Fluid Mech..

[28] Bouzidi M, Firdaouss M, Lallemand P (2001). Momentum transfer of a Boltzmann-lattice fluid with boundaries. Phys. Fluids.

[29] Wright DW, Richardson RA, Edeling W, Lakhlili J, Sinclair RC, Jancauskas V, Suleimenova D, Bosak B, Kulczewski M, Piontek T, Kopta P, Chirca I, Arabnejad H, Luk OO, Hoenen O, Wȩglarz J, Crommelin D, Groen D, Coveney PV (2020). Building confidence in simulation: Applications of Easy VVUQ. Adv. Theory Simulat..

[30] Richardson RA, Wright DW, Edeling W, Jancauskas V, Lakhlili J, Coveney PV (2020). EasyVVUQ: A library for verification, validation and uncertainty quantification in high performance computing. J. Open Res. Softw..

[31] UCL-CCS. EasyVVUQ. https://github.com/UCL-CCS/EasyVVUQ. (2022).

[32] VECMA. VECMA Toolkit, January. https://www.vecma-toolkit.eu/. (2022).

[33] SEAVEA. SEAVEA Toolkit, June. https://www.seavea-project.org/seaveatk/. (2022).

[34] McCullough JWS, Coveney PV (2021). High fidelity blood flow in a patient-specific arteriovenous fistula. Sci. Rep..

[35] Falcucci G, Amati G, Fanelli P, Krastev VK, Polverino G, Porfiri M, Succi S (2021). Extreme flow simulations reveal skeletal adaptations of deep-sea sponges. Nature.

[36] Falcucci G, Polverino G, Porfiri M, Amati G, Fanelli P, Krastev VK, Succi S (2022). Reply to: Models of flow through sponges must consider the sponge tissue. Nature.

