# Supplementary material for: Uncertainty quantification of the lattice Boltzmann method focussing on studies of human-scale vascular blood flow
Source: Sci Rep. 2024 May 17;14:11317. doi: 10.1038/s41598-024-61708-w (PMC11101457; doi:10.1038/s41598-024-61708-w)
Supplement: Supplementary file 1 — Supplementary Information. [file 41598_2024_61708_MOESM1_ESM.pdf]

Uncertainty quantification of the lattice Boltzmann method focussing on studies of human-scale vascular blood flow

Jon W.S. McCullough[1] and Peter V. Coveney*[1,2,3]

[1] *Centre for Computational Science, Department of Chemistry, University College London, UK*
[2] *Centre for Advanced Research Computing, University College London, UK*
[3] *Informatics Institute, University of Amsterdam, Netherlands*

April 4, 2024

1 Supplementary Information

Supplementary Figures 1 and 2 are larger presentations of that provided in Figure 2 of the main manuscript. The main channel is presented in Supplementary Figure 1 and the results for the side branch are in Supplementary Figure 2.

Supplementary Figures 3 - 7 illustrate how the analysis of the results presented in Figures 2 and 3 of the main manuscript vary with the order of the polynomial chaos expansion and that a converged state for Sobol indices is not reached here.

Supplementary Figures 8 and 9 are larger presentations of those provided in Figure 4 of the main manuscript. Supplementary Figures 10 - 13 present the flow results from the coarse domain under the cases of boundary condition variation discussed in the main manuscript.

1.1 High pressure cases

Supplementary Figures 14 - 16 present the flow results from the coarse domain under the first three cases of boundary condition variation discussed in the main manuscript with pressure conditions a factor of 100 higher. Supplementary Figures 18 - 21 present these higher pressure cases in the fine domain for all four boundary condition variations. The coefficients of variation calculated for these cases are presented in Supplementary Table 1.

In Supplementary Figures 14 and 15, where only one of the pressure outlets is being varied, it can be seen that the mean value of this changing pressure is the dominating factor in the variation of both the pressure and velocity quantities of interest. The area of exception is the velocity in the main channel prior to the bifurcation. Here the inlet velocity is the only factor influencing the measured quantity. Beyond the bifurcation, the impact of the inlet velocity is negligible.

When only the pressure outlets are being changed (Supplementary Figure 16), a high degree of symmetry between the analysed parameters is recorded. This would appear to indicate that for the chosen geometric configuration, the asymmetry of the domain does not impose a significant impact. This is potentially related to the flow distance between the point of observation and the pressure outlets being the same in this case. Before the bifurcation, the small variation of velocity within the channel is dominated by the amplitude of the oscillating boundary conditions. Beyond the bifurcation the combined effects of the chosen boundary conditions describes approximately 75% of the changes in velocity. The remaining 25% is almost fully accounted for by the mean pressure values suggesting that it is the combination of these that is also influential.

In the combined boundary case (Supplementary Figure 17) also records a combination of the observed statistical results. Before the bifurcation, the inlet velocity is solely responsible for the

Study	$CV(\vec{q})$	$CV\left(\vec{\xi}\right)$	CVR
Coarse Bifurcation BC1	0.5989	0.5004	1.1968
Coarse Bifurcation BC2	0.5831	0.5004	1.1653
Coarse Bifurcation BC3	0.7227	0.5774	1.2517
Coarse Bifurcation BC4	0.7410	0.5774	1.2835
Fine Bifurcation BC1	0.5996	0.5004	1.1983
Fine Bifurcation BC2	0.5824	0.5004	1.1639
Fine Bifurcation BC3	0.7302	0.5774	1.2648
Fine Bifurcation BC4	0.7544	0.5774	1.3067

Supplementary Table 1: Coefficients of variation of input parameters and quantities of interest as generated by the LBM implemented within HemeLB for the high pressure boundary condition campaigns.

variation in the domain and less than 10% of pressure variance. The mean pressure of the outlet boundaries, either singularly or in combination, are responsible for over 90% of the change in pressure distribution throughout the whole domain. Beyond the bifurcation they are also responsible for the variation in velocity and mostly in a higher order combination. The distribution of velocity increases significantly once it becomes driven by the pressure boundary conditions.

1.2 Forearm arteries

In Supplementary Figure 22, the normal distribution of the mean and maximum velocities observed in the personalised vessels can be observed. This data has been sampled based on a third-order polynomial chaos expansion of the observed simulation data.

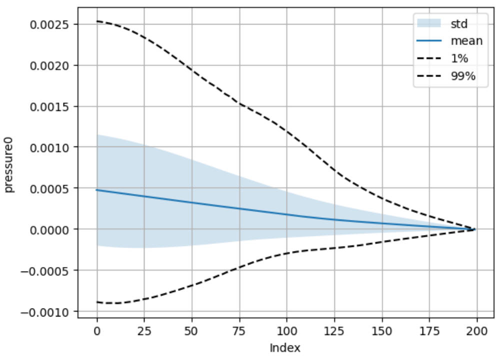

(a) Coarse - Pressure distribution

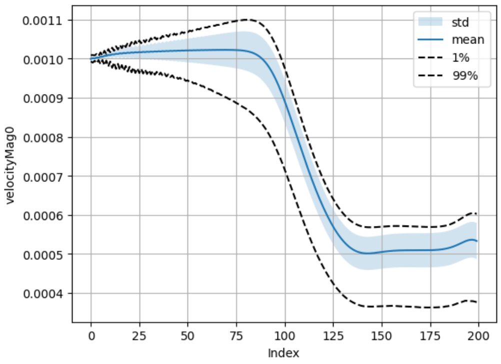

(b) Coarse - Velocity distribution

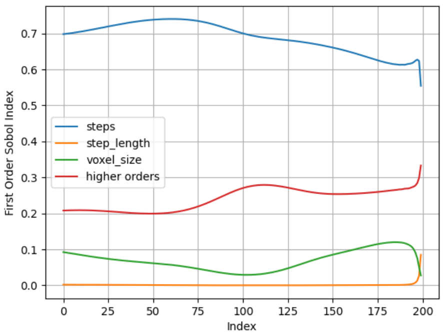

(c) Coarse - Pressure Sobol indices

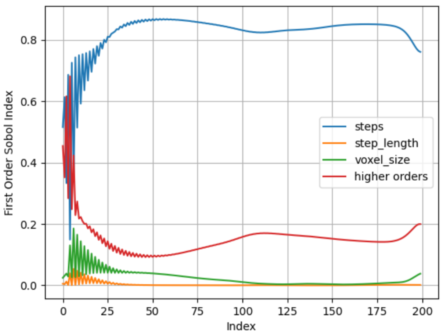

(d) Coarse - Velocity Sobol indices

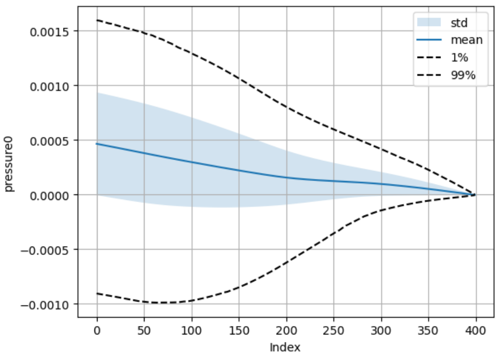

(e) Fine - Pressure distribution

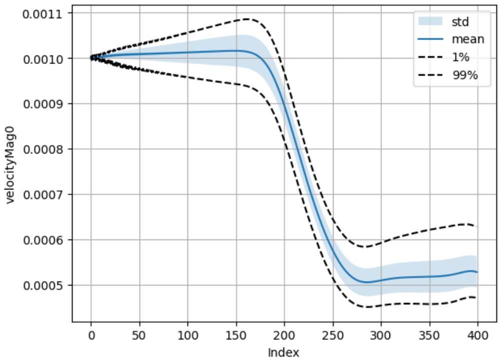

(f) Fine - Velocity distribution

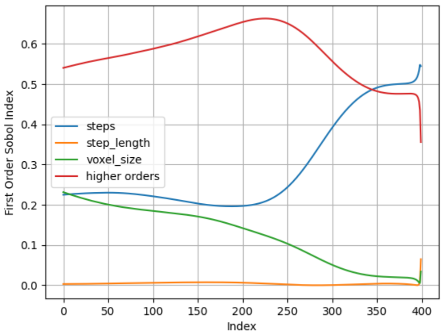

(g) Fine - Pressure Sobol indices

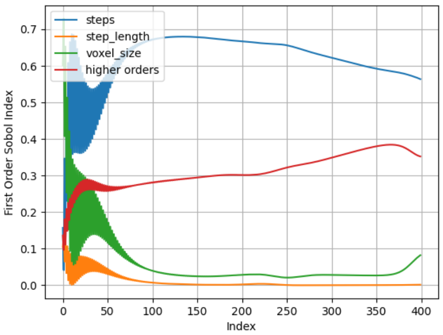

(h) Fine - Velocity Sobol indices

Supplementary Figure 1: Uncertainty analysis of the pressure and velocity within the main channel coarse and fine bifurcation geometries determined with a third-order polynomial chaos expansion. Algorithmic parameters of the LBM have been varied here with constant boundary conditions.

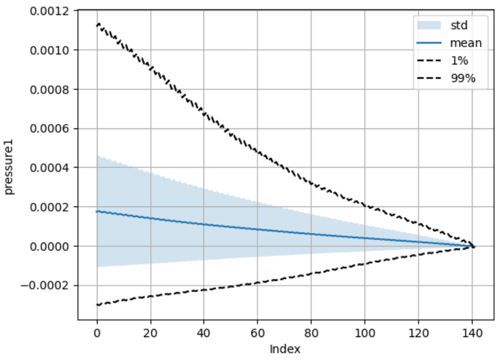

(a) Coarse - Pressure distribution

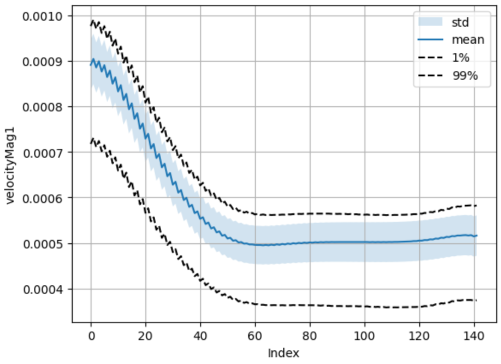

(b) Coarse - Velocity distribution

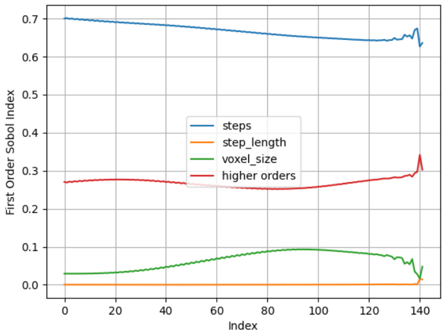

(c) Coarse - Pressure Sobol indices

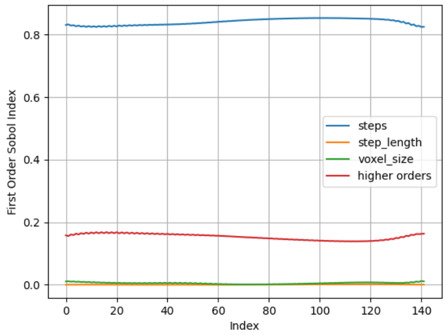

(d) Coarse - Velocity Sobol indices

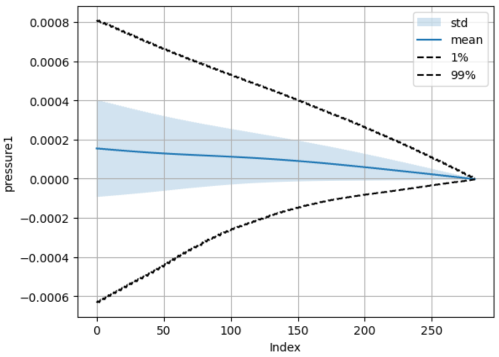

(e) Fine - Pressure distribution

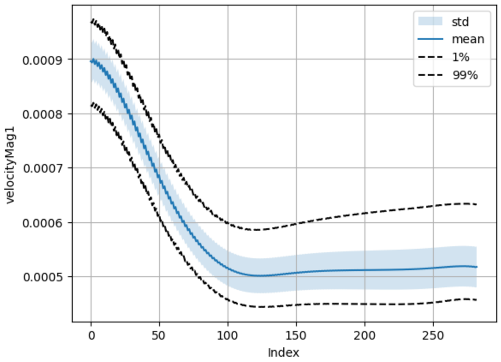

(f) Fine - Velocity distribution

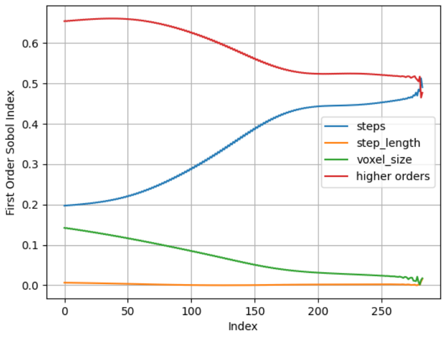

(g) Fine - Pressure Sobol indices

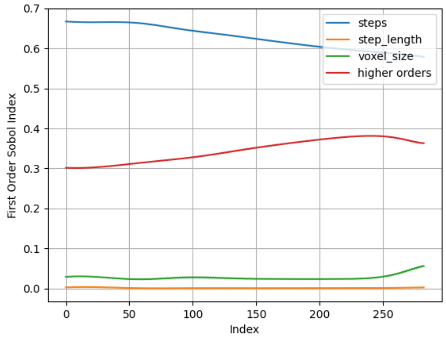

(h) Fine - Velocity Sobol indices

Supplementary Figure 2: Uncertainty analysis of the pressure and velocity within the side branch of the coarse and fine bifurcation geometries determined with a third-order polynomial chaos expansion. Algorithmic parameters of the LBM have been varied here with constant boundary conditions.

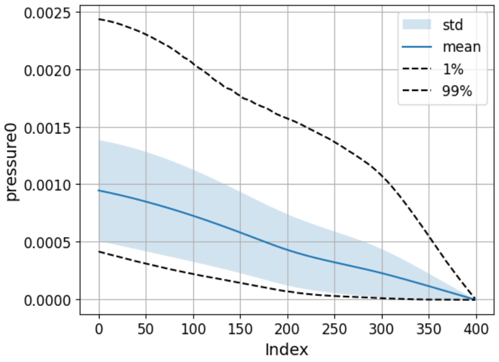

(a) Main - Pressure distribution

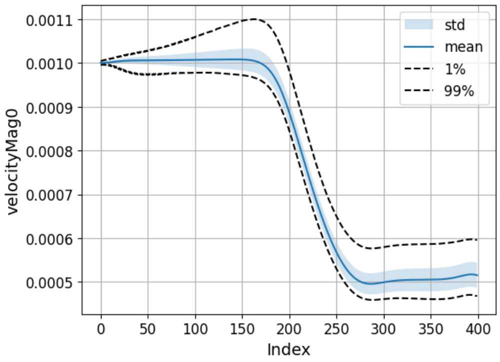

(b) Main - Velocity distribution

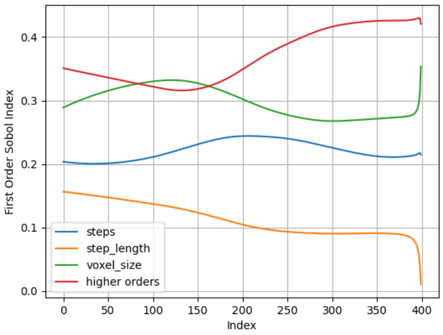

(c) Main - Pressure Sobol indices

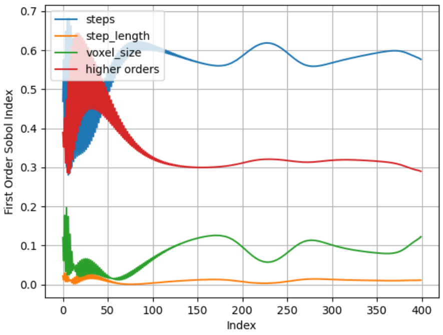

(d) Main - Velocity Sobol indices

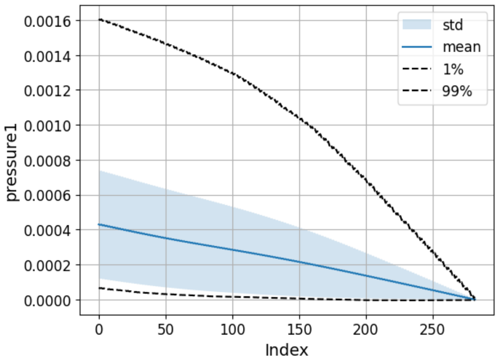

(e) Branch - Pressure distribution

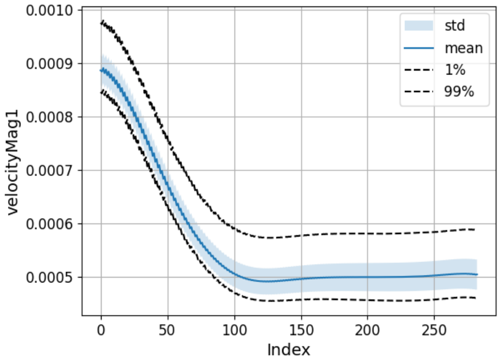

(f) Branch - Velocity distribution

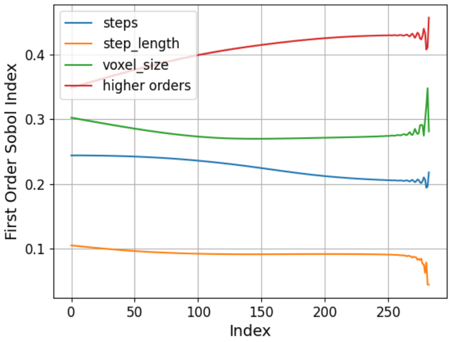

(g) Branch - Pressure Sobol indices

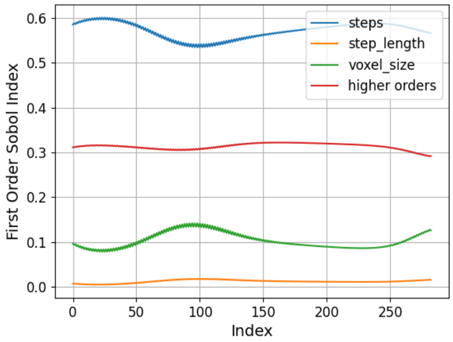

(h) Branch - Velocity Sobol indices

Supplementary Figure 3: Uncertainty and parameter sensitivity analysis of the pressure and velocity within the bifurcation geometry determined with a fourth-order polynomial chaos expansion. Algorithmic parameters of the LBM have been varied here with constant boundary conditions.

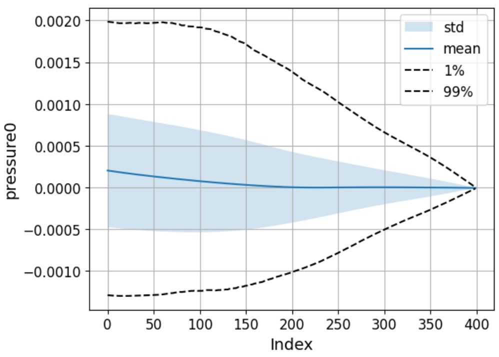

(a) Main - Pressure distribution

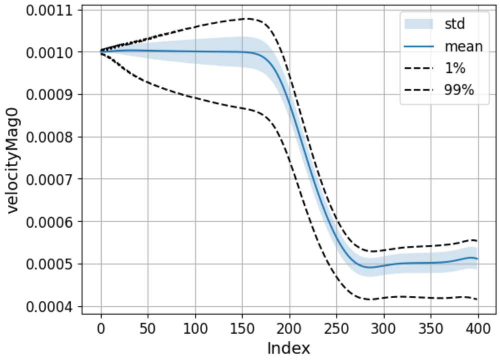

(b) Main - Velocity distribution

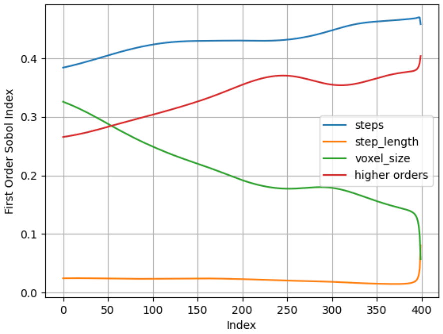

(c) Main - Pressure Sobol indices

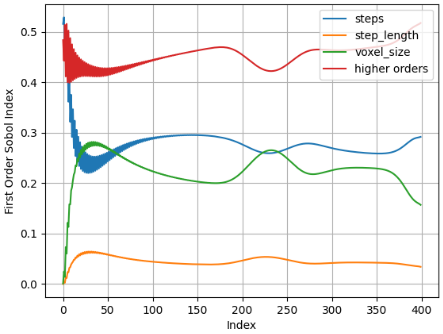

(d) Main - Velocity Sobol indices

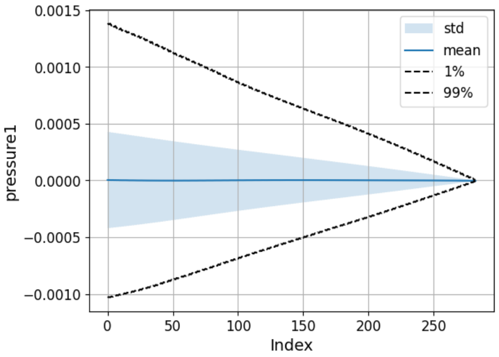

(e) Branch - Pressure distribution

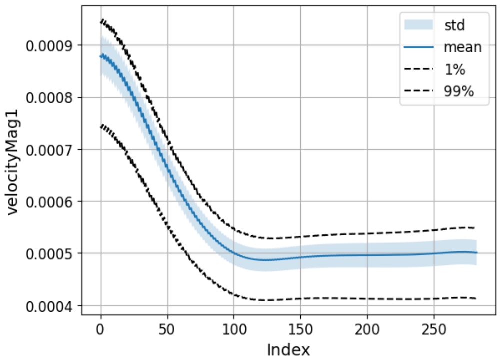

(f) Branch - Velocity distribution

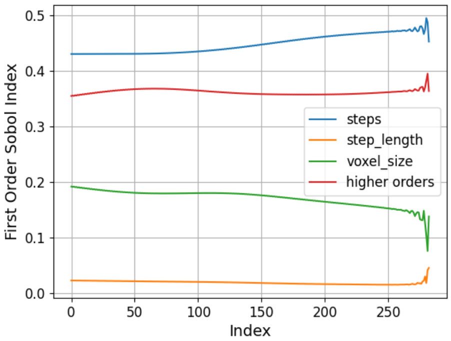

(g) Branch - Pressure Sobol indices

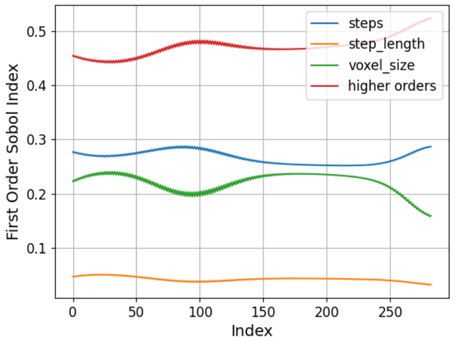

(h) Branch - Velocity Sobol indices

Supplementary Figure 4: Uncertainty and parameter sensitivity analysis of the pressure and velocity within the bifurcation geometry determined with a fifth-order polynomial chaos expansion. Algorithmic parameters of the LBM have been varied here with constant boundary conditions.

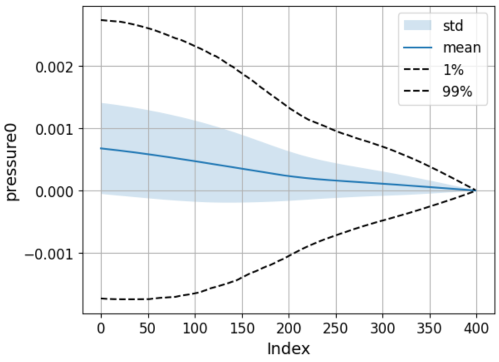

(a) Main - Pressure distribution

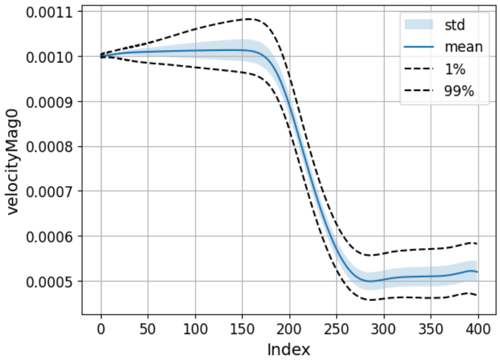

(b) Main - Velocity distribution

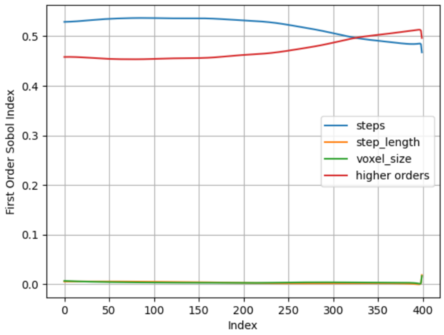

(c) Main - Pressure Sobol indices

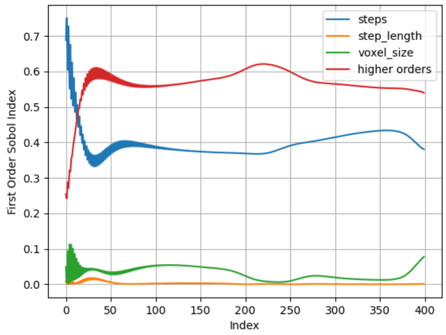

(d) Main - Velocity Sobol indices

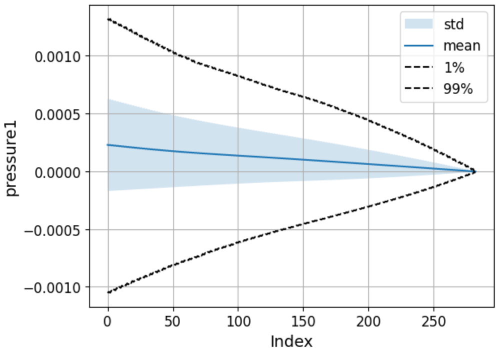

(e) Branch - Pressure distribution

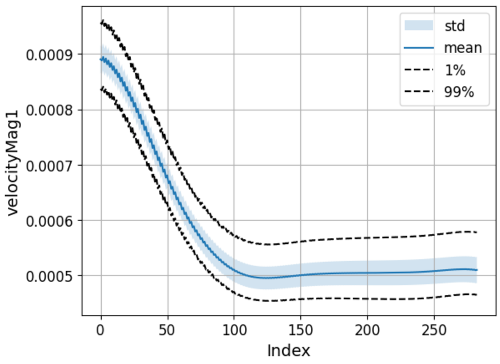

(f) Branch - Velocity distribution

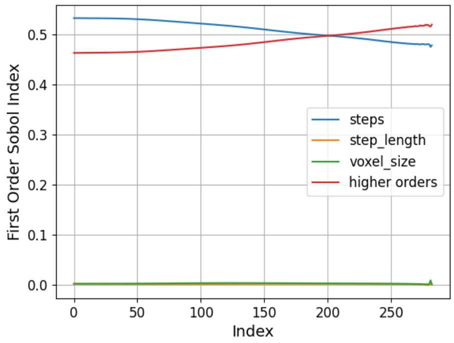

(g) Branch - Pressure Sobol indices

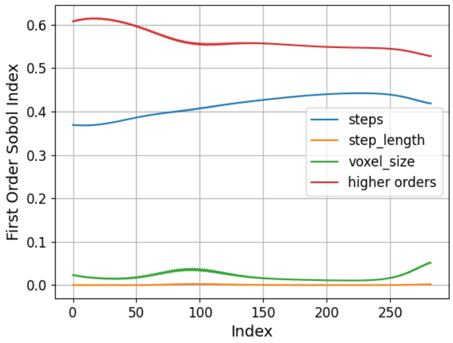

(h) Branch - Velocity Sobol indices

Supplementary Figure 5: Uncertainty and parameter sensitivity analysis of the pressure and velocity within the bifurcation geometry determined with a sixth-order polynomial chaos expansion. Algorithmic parameters of the LBM have been varied here with constant boundary conditions.

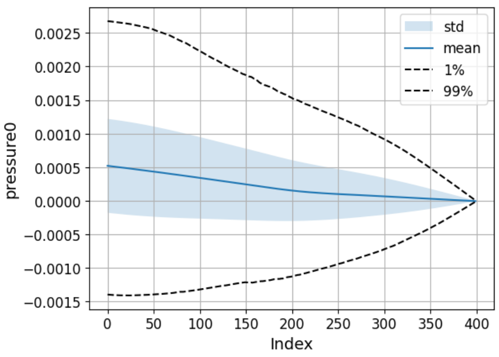

(a) Main - Pressure distribution

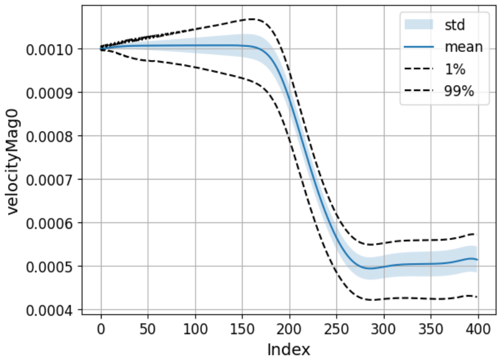

(b) Main - Velocity distribution

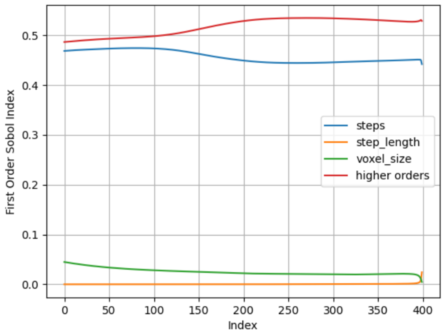

(c) Main - Pressure Sobol indices

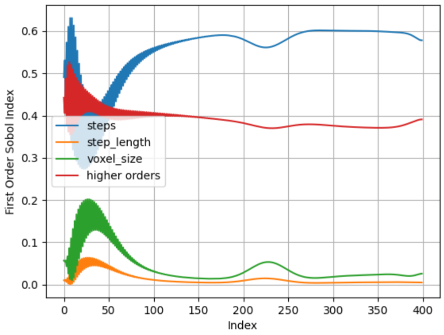

(d) Main - Velocity Sobol indices

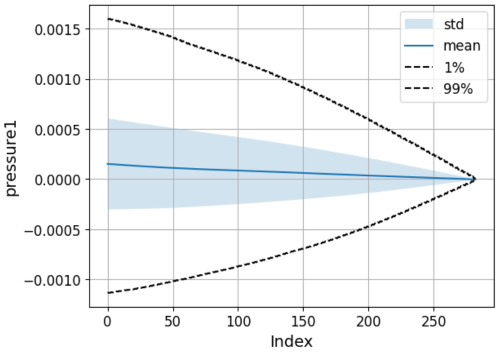

(e) Branch - Pressure distribution

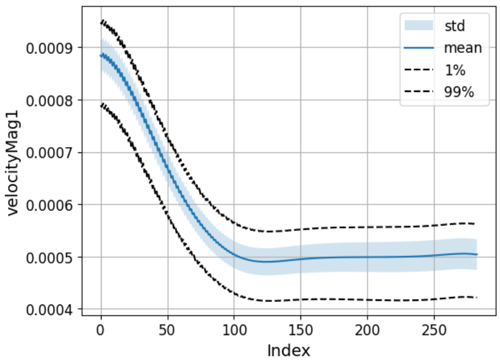

(f) Branch - Velocity distribution

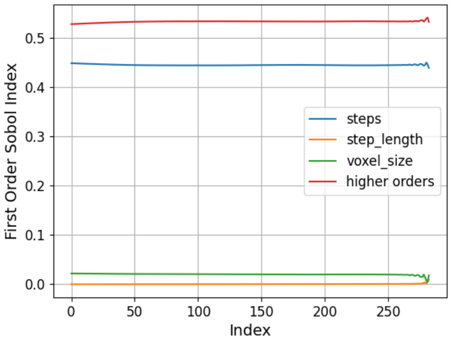

(g) Branch - Pressure Sobol indices

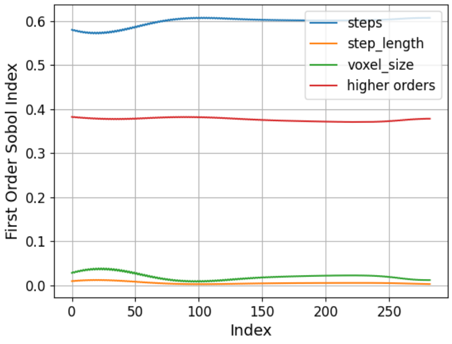

(h) Branch - Velocity Sobol indices

Supplementary Figure 6: Uncertainty and parameter sensitivity analysis of the pressure and velocity within the bifurcation geometry determined with a seventh-order polynomial chaos expansion. Algorithmic parameters of the LBM have been varied here with constant boundary conditions.

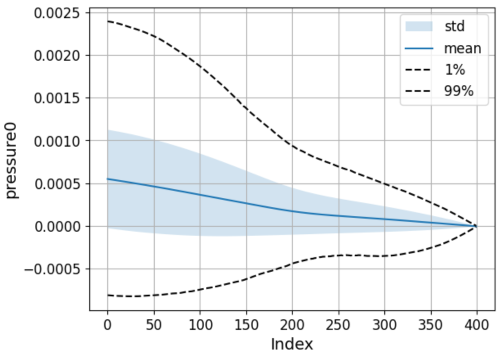

(a) Main - Pressure distribution

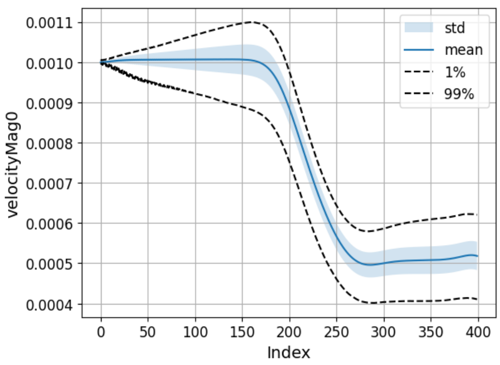

(b) Main - Velocity distribution

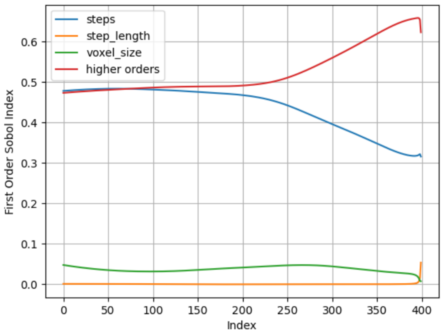

(c) Main - Pressure Sobol indices

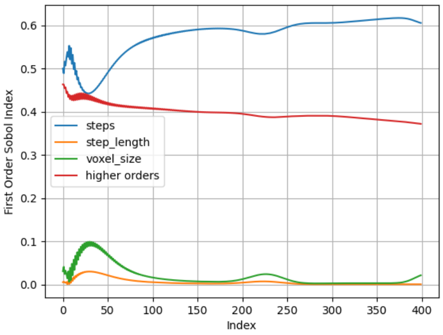

(d) Main - Velocity Sobol indices

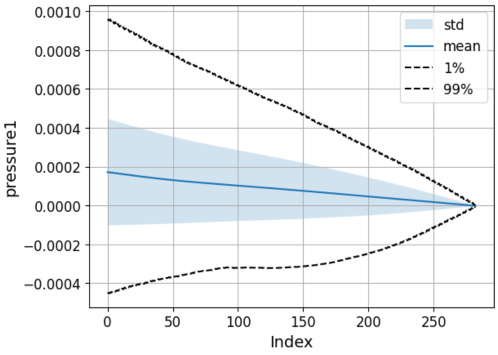

(e) Branch - Pressure distribution

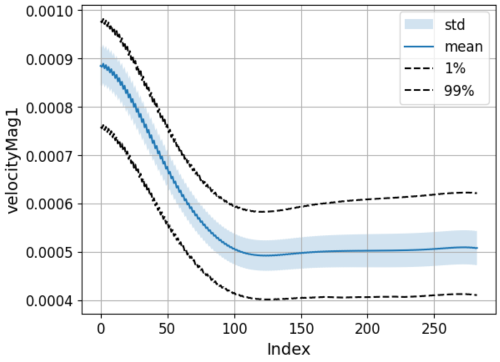

(f) Branch - Velocity distribution

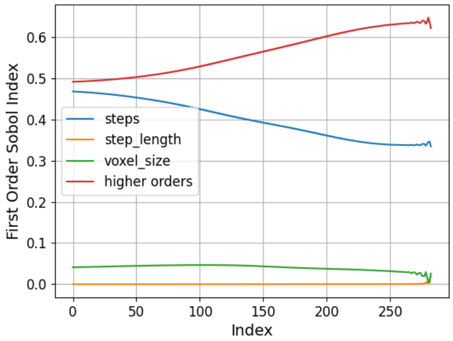

(g) Branch - Pressure Sobol indices

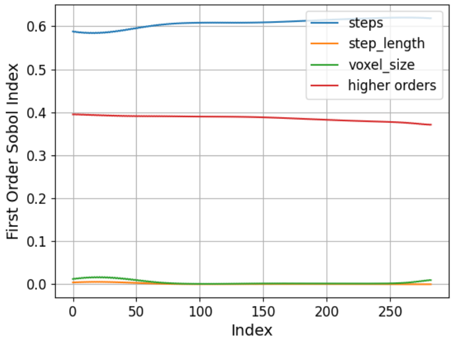

(h) Branch - Velocity Sobol indices

Supplementary Figure 7: Uncertainty and parameter sensitivity analysis of the pressure and velocity within the bifurcation geometry determined with an eighth-order polynomial chaos expansion. Algorithmic parameters of the LBM have been varied here with constant boundary conditions.

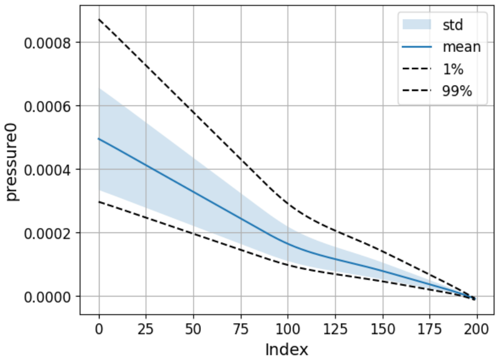

(a) Coarse - Pressure distribution

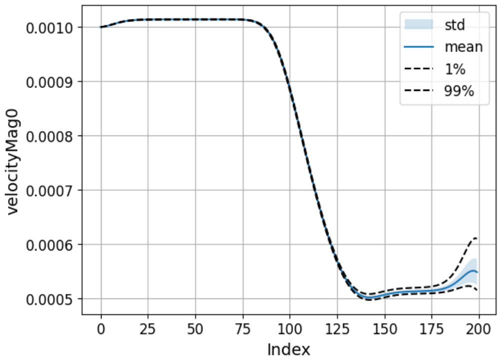

(b) Coarse - Velocity distribution

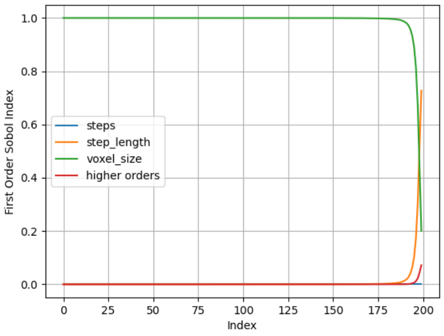

(c) Coarse - Pressure Sobol indices

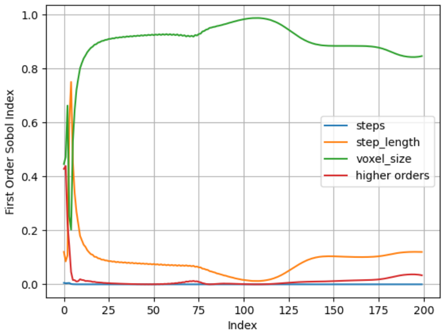

(d) Coarse - Velocity Sobol indices

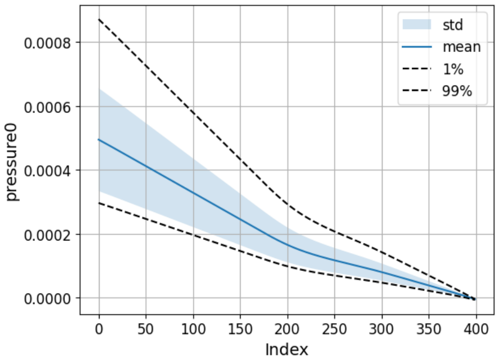

(e) Fine - Pressure distribution

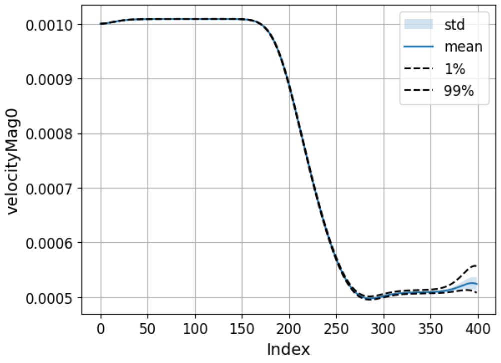

(f) Fine - Velocity distribution

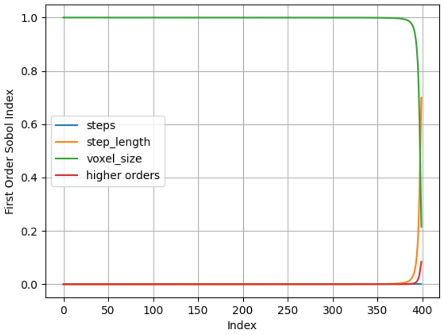

(g) Fine - Pressure Sobol indices

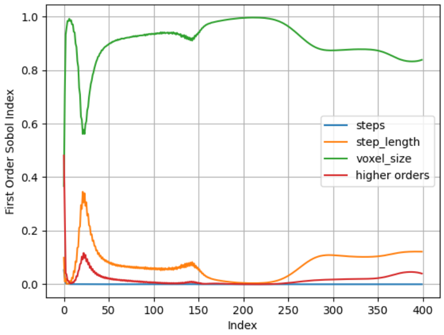

(h) Fine - Velocity Sobol indices

Supplementary Figure 8: Uncertainty analysis of the pressure and velocity within the main channel coarse and fine bifurcation geometries determined with a third-order polynomial chaos expansion. Algorithmic parameters of the LBM have been varied here with constant boundary conditions.

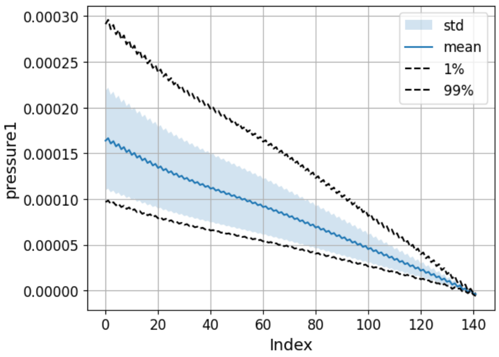

(a) Coarse - Pressure distribution

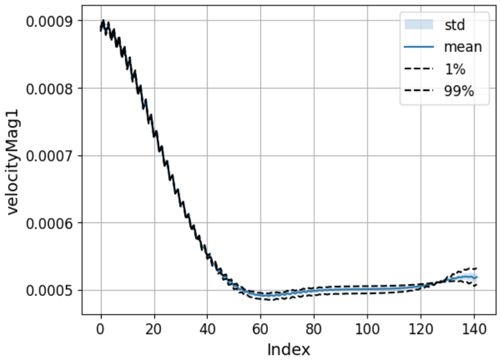

(b) Coarse - Velocity distribution

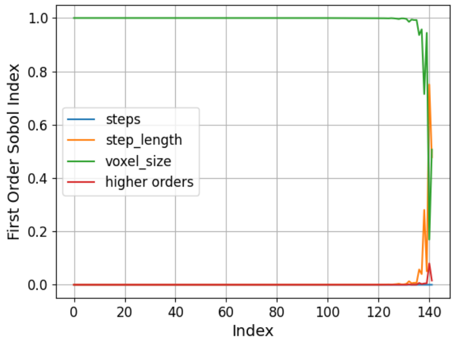

(c) Coarse - Pressure Sobol indices

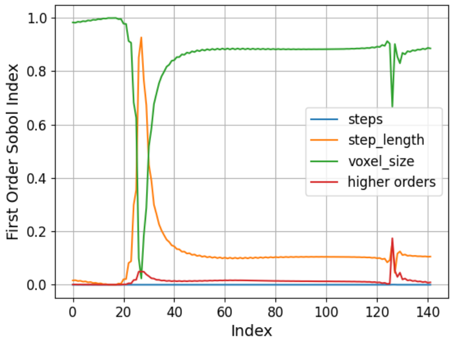

(d) Coarse - Velocity Sobol indices

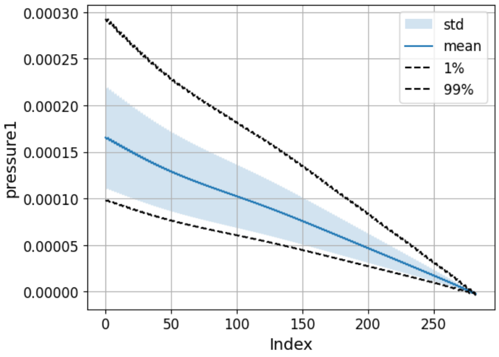

(e) Fine - Pressure distribution

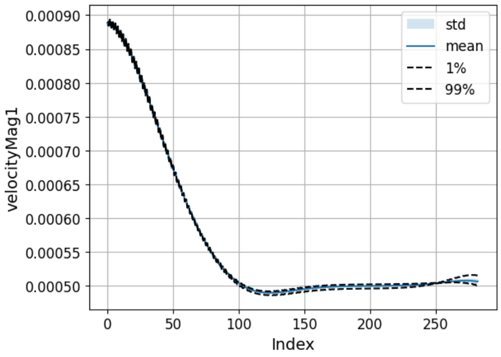

(f) Fine - Velocity distribution

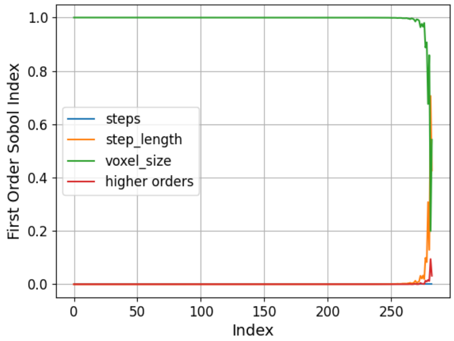

(g) Fine - Pressure Sobol indices

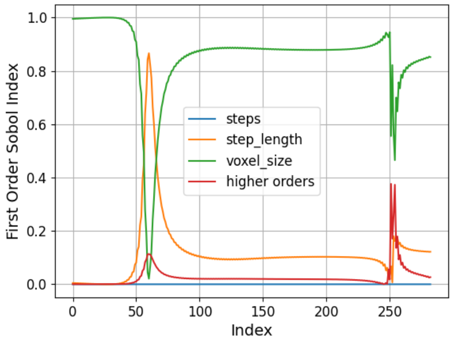

(h) Fine - Velocity Sobol indices

Supplementary Figure 9: Uncertainty analysis of the pressure and velocity within the side branch of the coarse and fine bifurcation geometries determined with a third-order polynomial chaos expansion. Algorithmic parameters of the LBM have been varied here with constant boundary conditions.

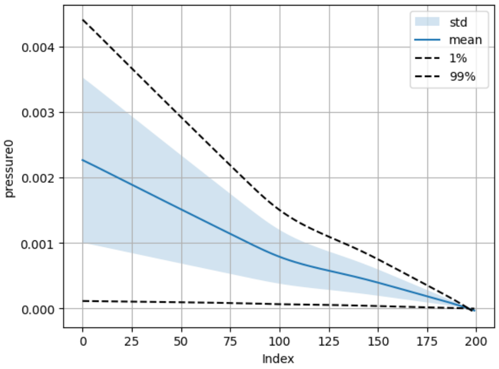

(a) Main - Pressure distribution

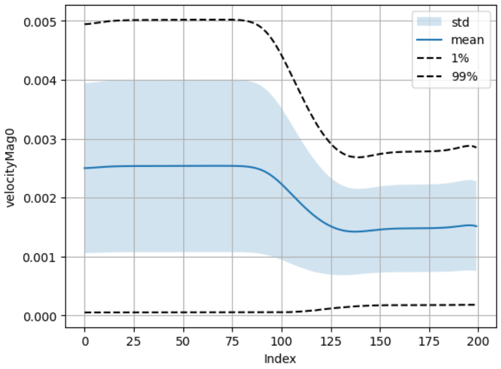

(b) Main - Velocity distribution

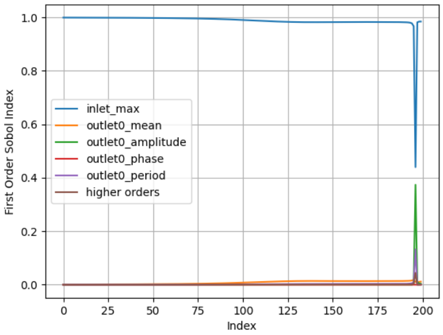

(c) Main - Pressure Sobol indices

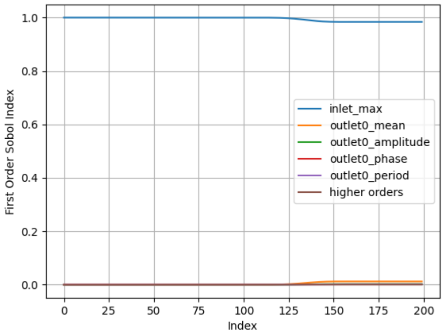

(d) Main - Velocity Sobol indices

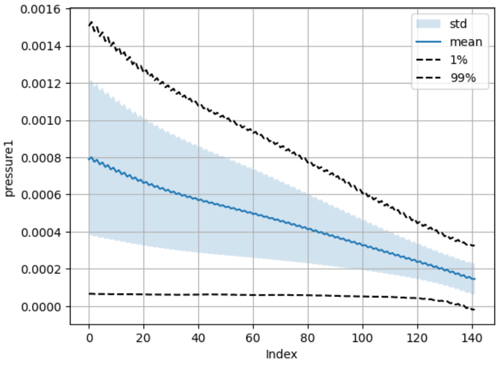

(e) Branch - Pressure distribution

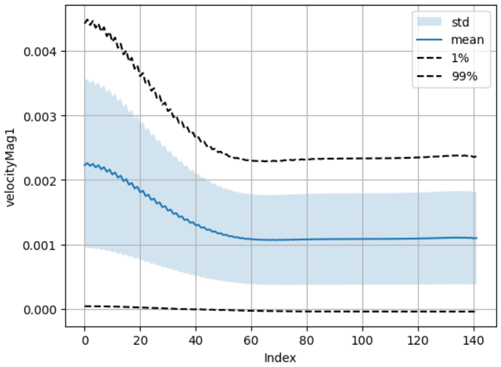

(f) Branch - Velocity distribution

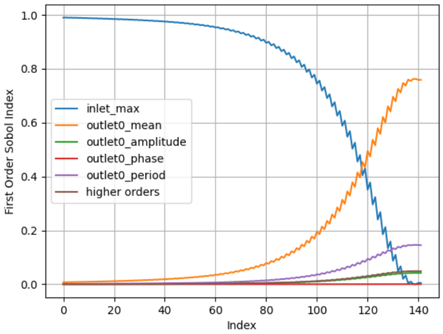

(g) Branch - Pressure Sobol indices

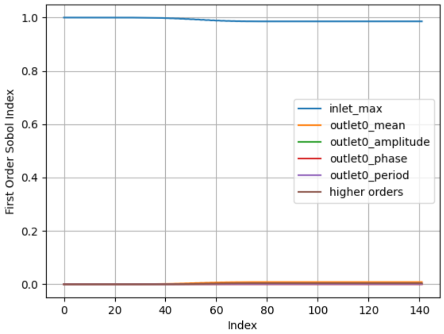

(h) Branch - Velocity Sobol indices

Supplementary Figure 10: Uncertainty analysis of the pressure and velocity within the coarse bifurcation geometries determined with a third-order polynomial chaos expansion. Boundary conditions consistent with Figure 4 of the main manuscript.

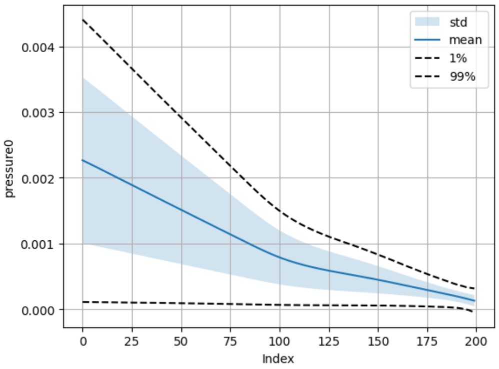

(a) Main - Pressure distribution

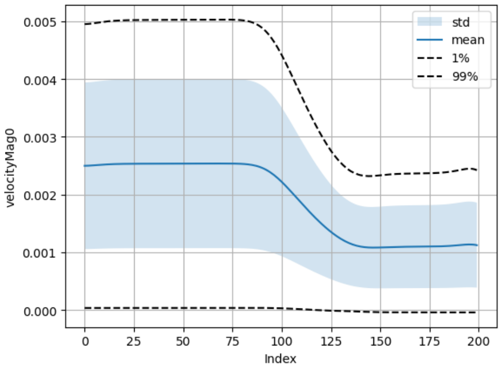

(b) Main - Velocity distribution

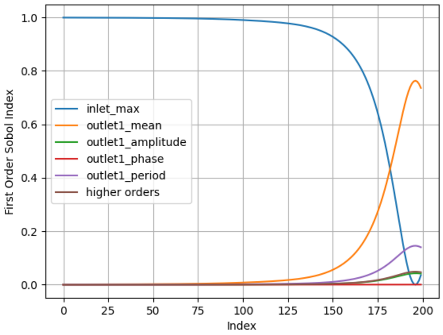

(c) Main - Pressure Sobol indices

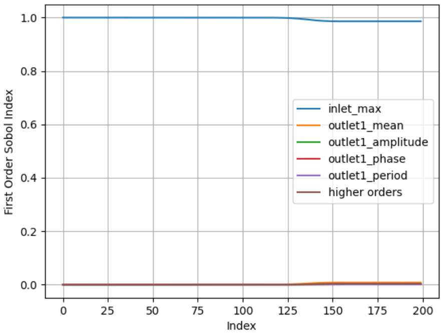

(d) Main - Velocity Sobol indices

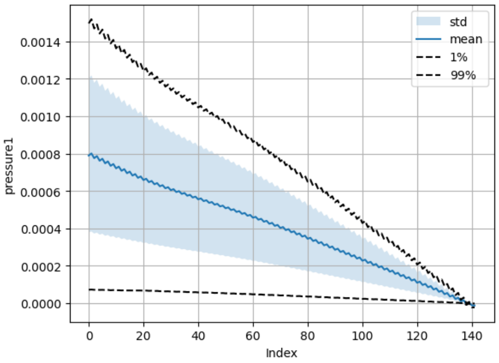

(e) Branch - Pressure distribution

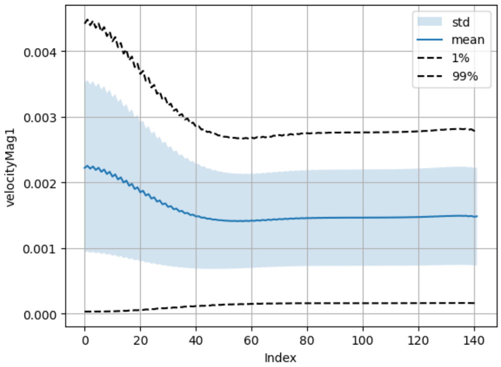

(f) Branch - Velocity distribution

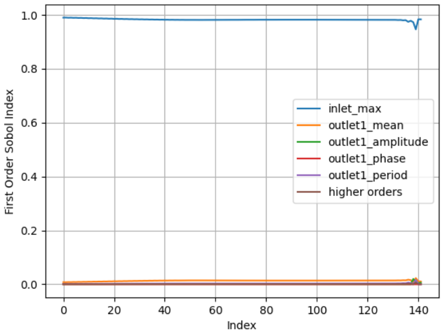

(g) Branch - Pressure Sobol indices

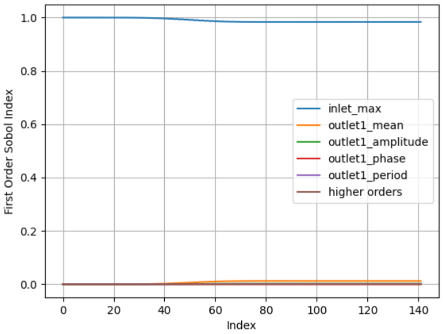

(h) Branch - Velocity Sobol indices

Supplementary Figure 11: Uncertainty analysis of the pressure and velocity within the coarse bifurcation geometries determined with a third-order polynomial chaos expansion. Boundary conditions consistent with Figure 5 of the main manuscript.

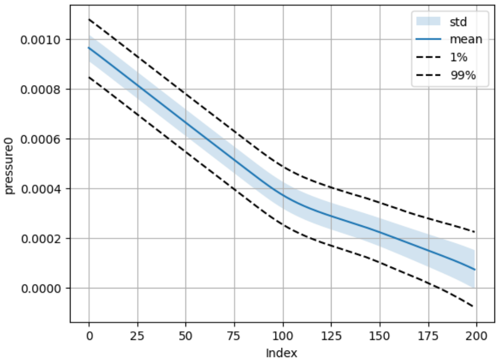

(a) Main - Pressure distribution

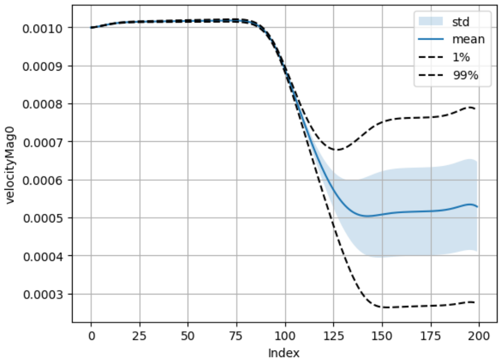

(b) Main - Velocity distribution

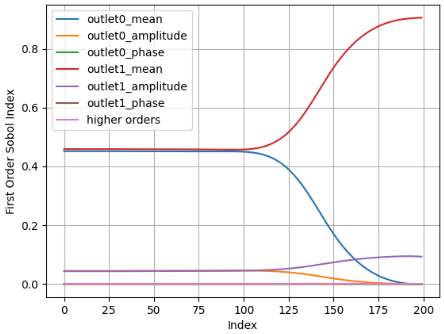

(c) Main - Pressure Sobol indices

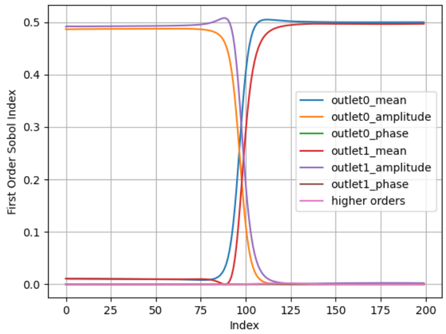

(d) Main - Velocity Sobol indices

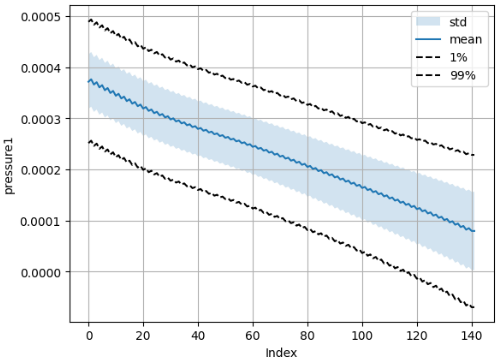

(e) Branch - Pressure distribution

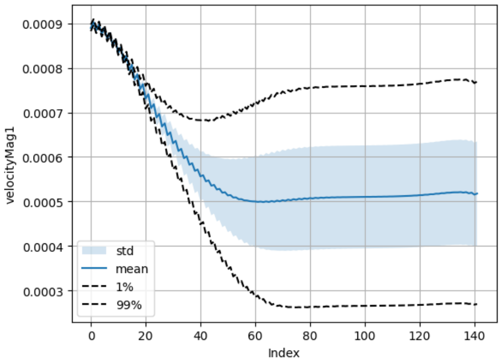

(f) Branch - Velocity distribution

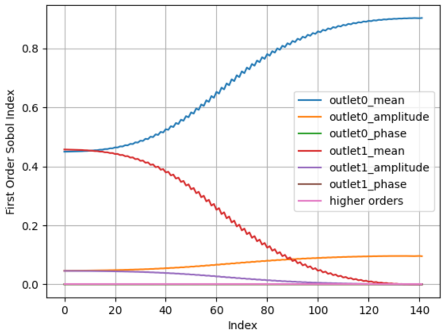

(g) Branch - Pressure Sobol indices

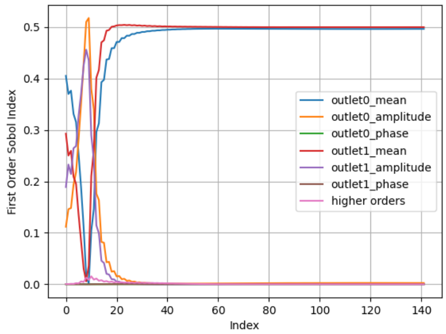

(h) Branch - Velocity Sobol indices

Supplementary Figure 12: Uncertainty analysis of the pressure and velocity within the coarse bifurcation geometries determined with a third-order polynomial chaos expansion. Boundary conditions consistent with Figure 6 of the main manuscript.

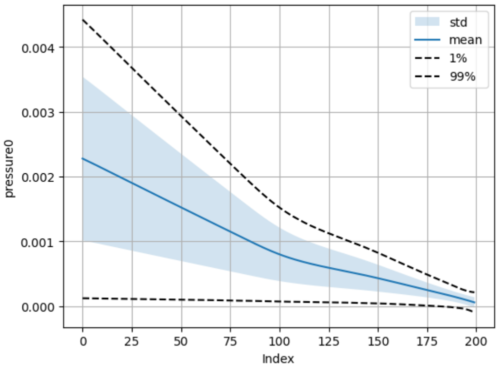

(a) Main - Pressure distribution

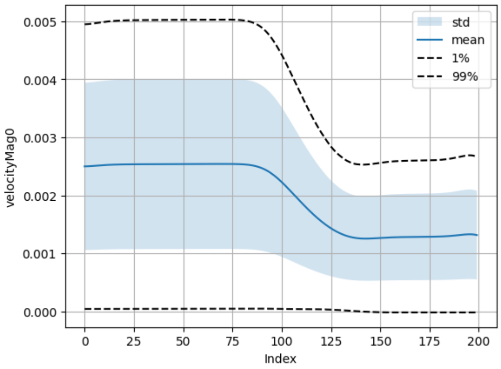

(b) Main - Velocity distribution

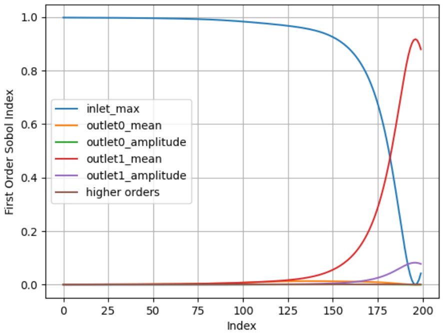

(c) Main - Pressure Sobol indices

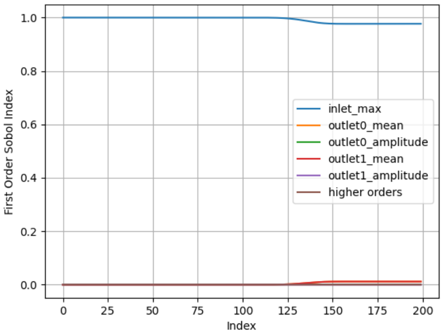

(d) Main - Velocity Sobol indices

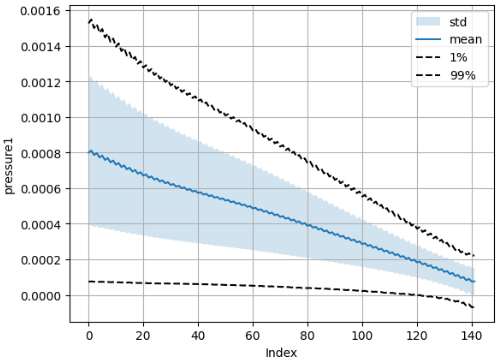

(e) Branch - Pressure distribution

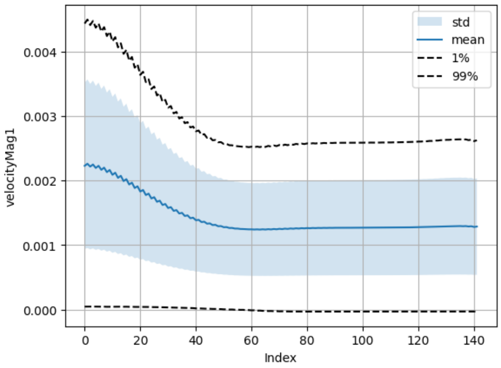

(f) Branch - Velocity distribution

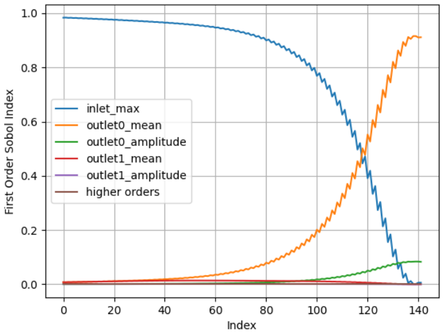

(g) Branch - Pressure Sobol indices

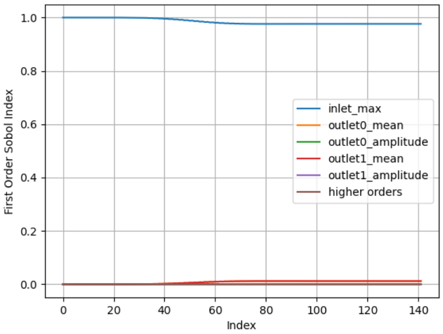

(h) Branch - Velocity Sobol indices

Supplementary Figure 13: Uncertainty analysis of the pressure and velocity within the coarse bifurcation geometries determined with a third-order polynomial chaos expansion. Boundary conditions consistent with Figure 7 of the main manuscript.

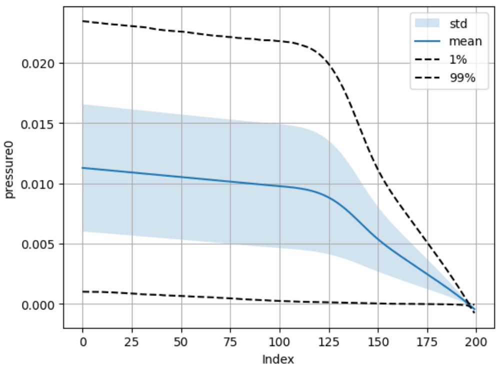

(a) Main - Pressure distribution

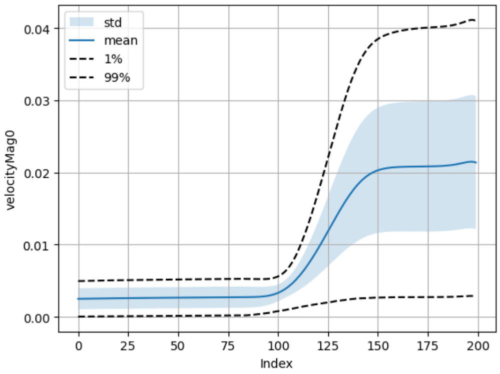

(b) Main - Velocity distribution

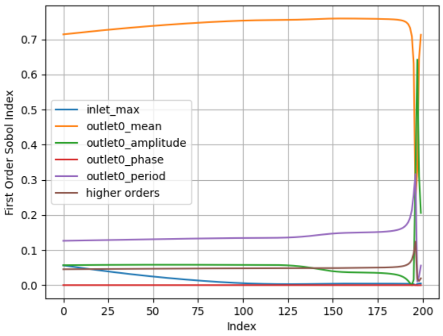

(c) Main - Pressure Sobol indices

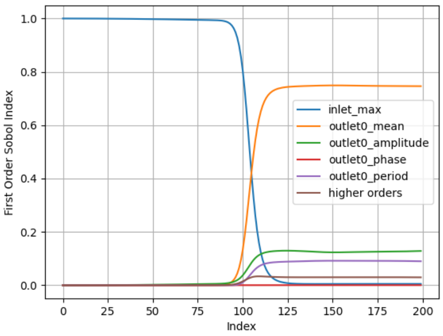

(d) Main - Velocity Sobol indices

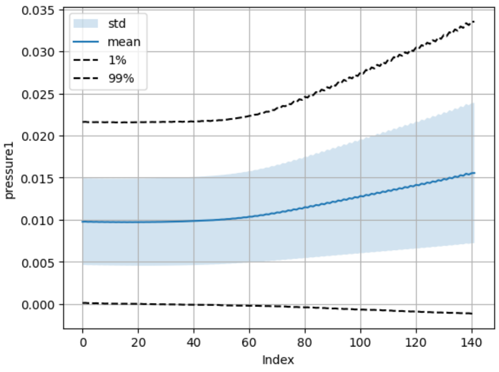

(e) Branch - Pressure distribution

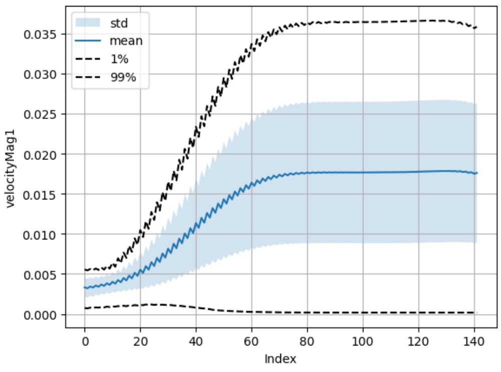

(f) Branch - Velocity distribution

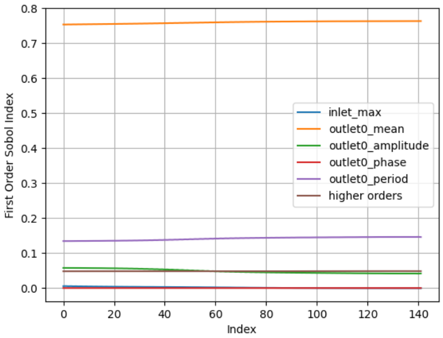

(g) Branch - Pressure Sobol indices

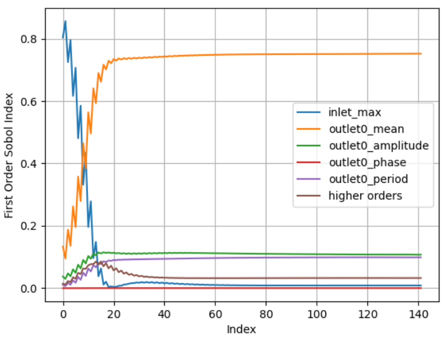

(h) Branch - Velocity Sobol indices

Supplementary Figure 14: Uncertainty analysis of the pressure and velocity within the coarse bifurcation geometries determined with a third-order polynomial chaos expansion (BC1, higher pressure).

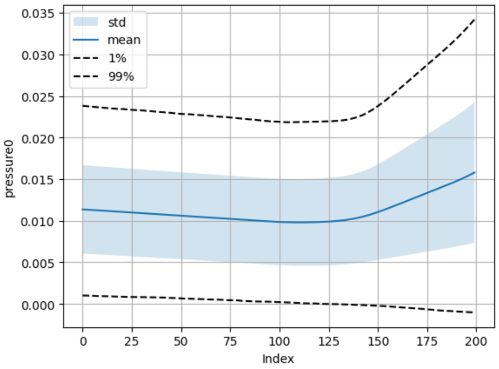

(a) Main - Pressure distribution

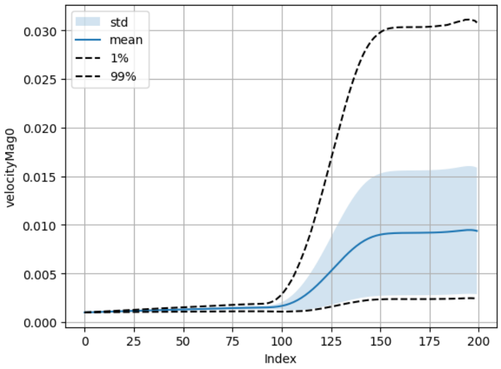

(b) Main - Velocity distribution

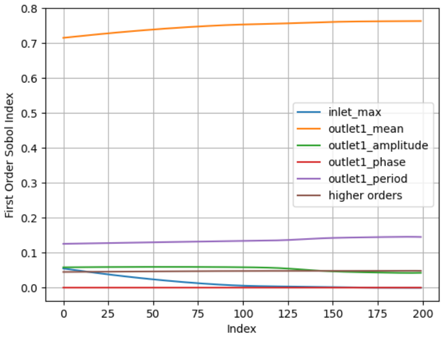

(c) Main - Pressure Sobol indices

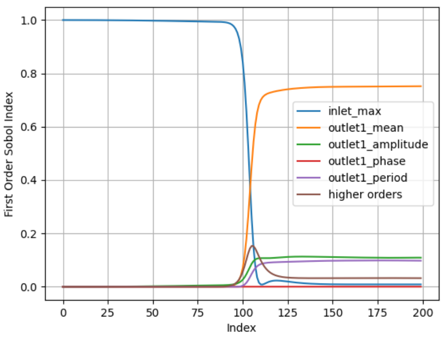

(d) Main - Velocity Sobol indices

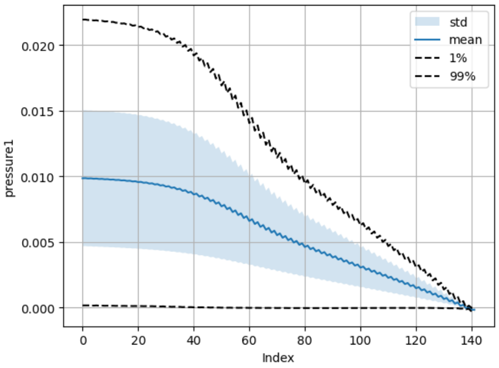

(e) Branch - Pressure distribution

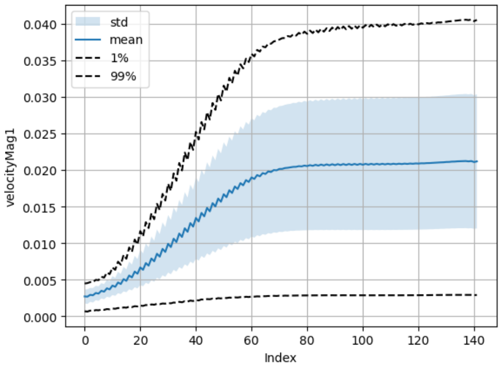

(f) Branch - Velocity distribution

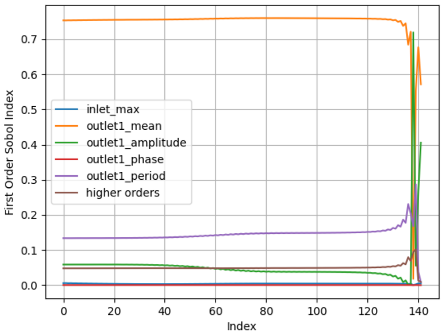

(g) Branch - Pressure Sobol indices

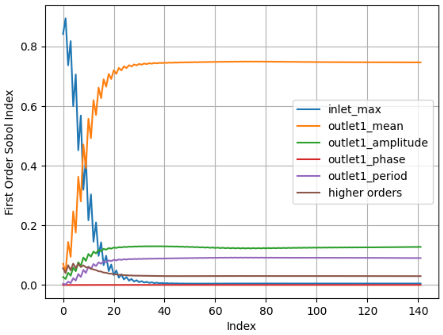

(h) Branch - Velocity Sobol indices

Supplementary Figure 15: Uncertainty analysis of the pressure and velocity within the coarse bifurcation geometries determined with a third-order polynomial chaos expansion (BC2, higher pressure).

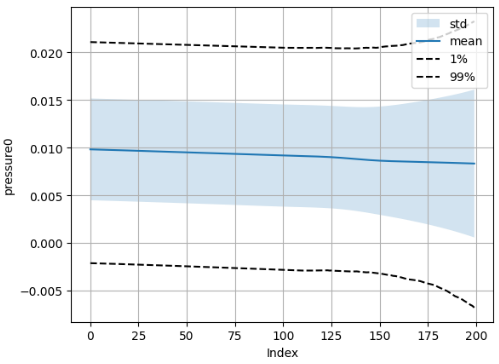

(a) Main - Pressure distribution

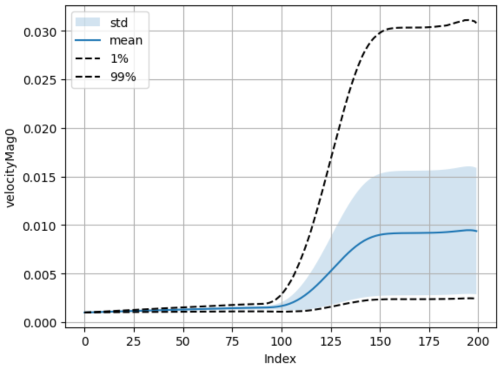

(b) Main - Velocity distribution

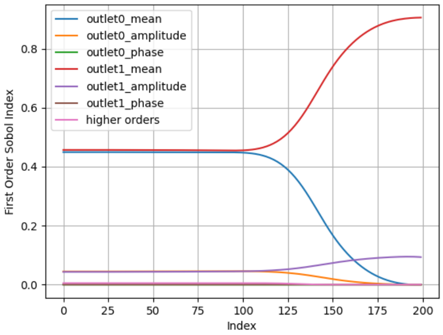

(c) Main - Pressure Sobol indices

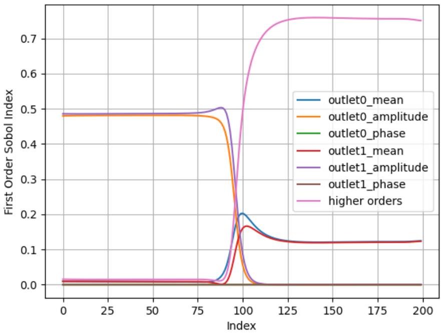

(d) Main - Velocity Sobol indices

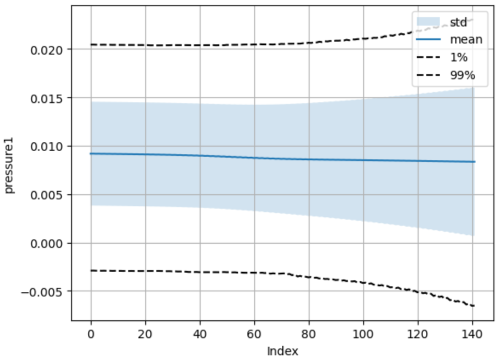

(e) Branch - Pressure distribution

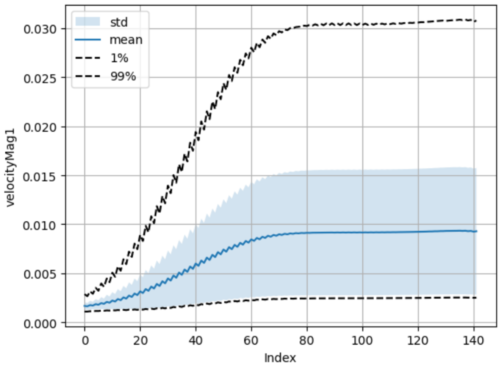

(f) Branch - Velocity distribution

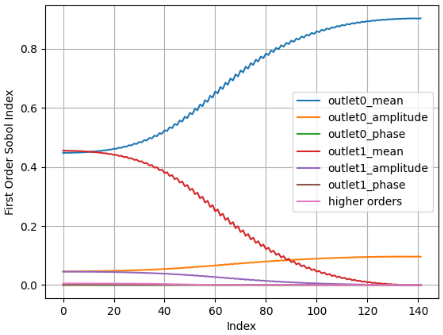

(g) Branch - Pressure Sobol indices

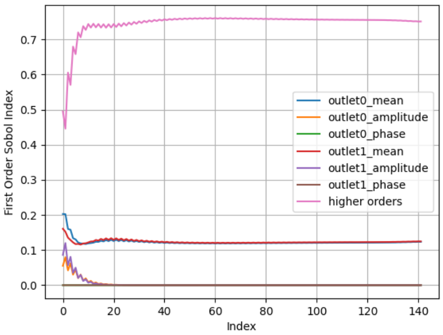

(h) Branch - Velocity Sobol indices

Supplementary Figure 16: Uncertainty analysis of the pressure and velocity within the coarse bifurcation geometries determined with a third-order polynomial chaos expansion (BC3, higher pressure).

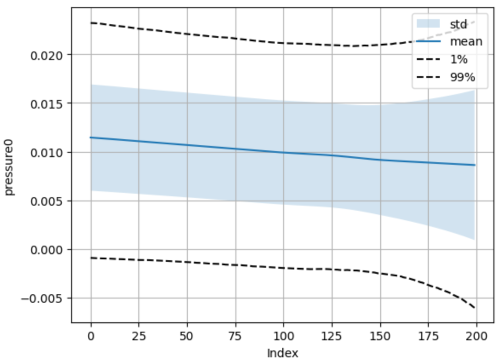

(a) Main - Pressure distribution

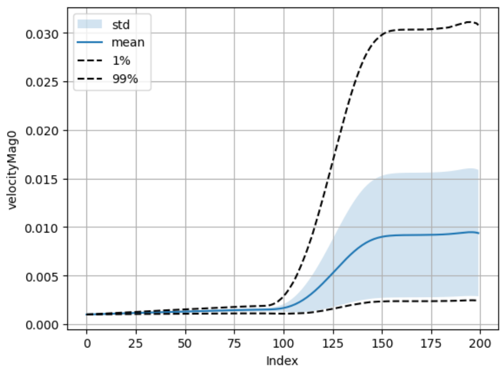

(b) Main - Velocity distribution

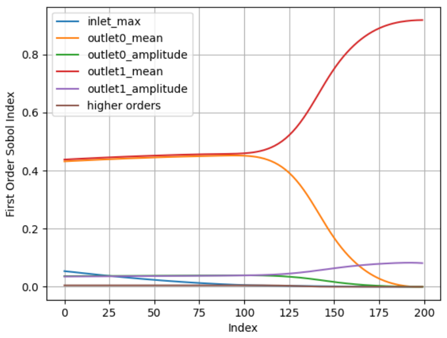

(c) Main - Pressure Sobol indices

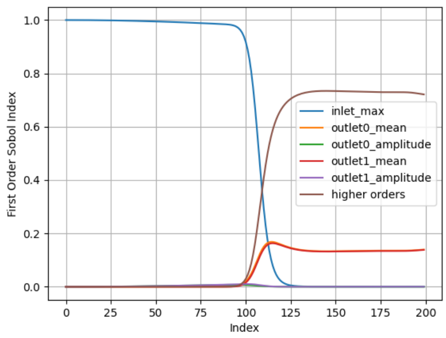

(d) Main - Velocity Sobol indices

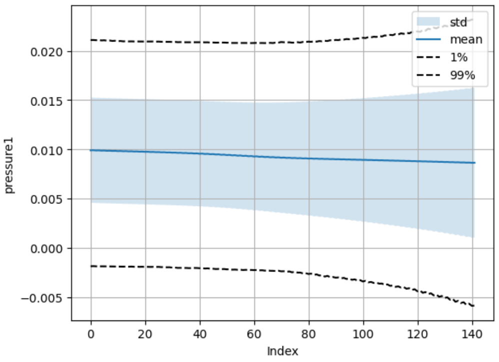

(e) Branch - Pressure distribution

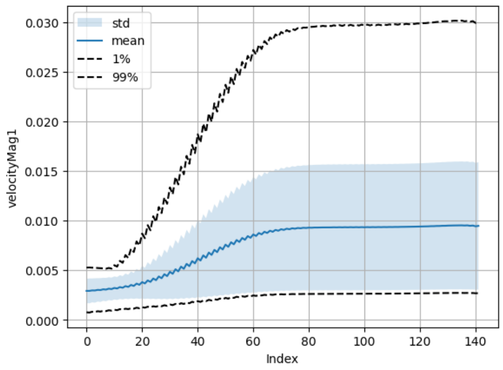

(f) Branch - Velocity distribution

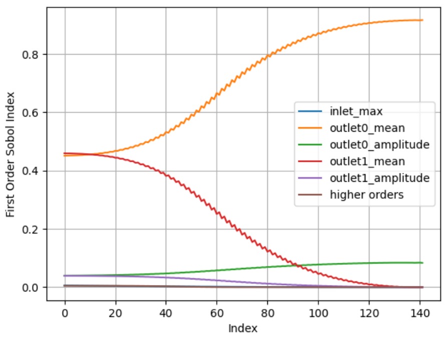

(g) Branch - Pressure Sobol indices

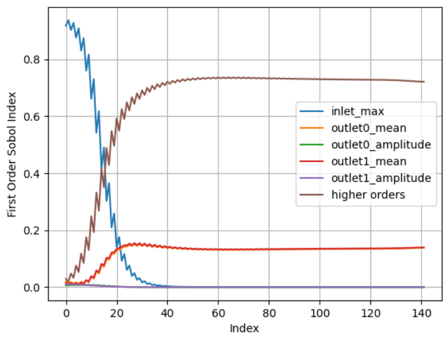

(h) Branch - Velocity Sobol indices

Supplementary Figure 17: Uncertainty analysis of the pressure and velocity within the coarse bifurcation geometries determined with a third-order polynomial chaos expansion (BC4, higher pressure).

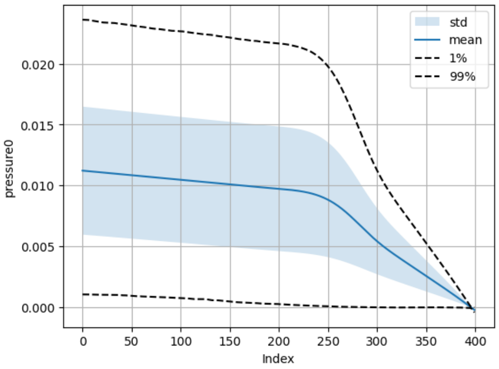

(a) Main - Pressure distribution

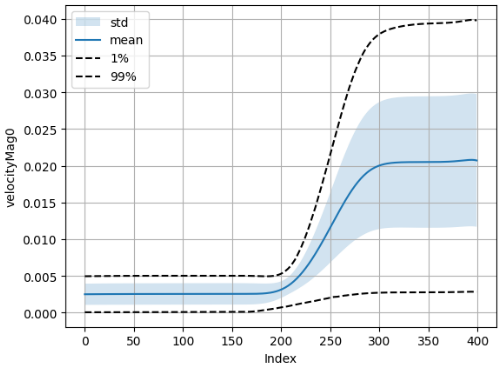

(b) Main - Velocity distribution

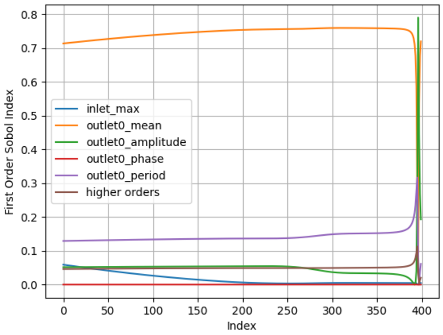

(c) Main - Pressure Sobol indices

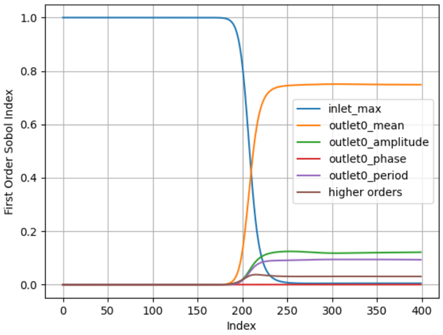

(d) Main - Velocity Sobol indices

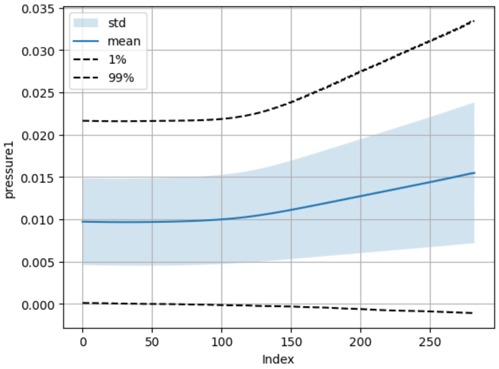

(e) Branch - Pressure distribution

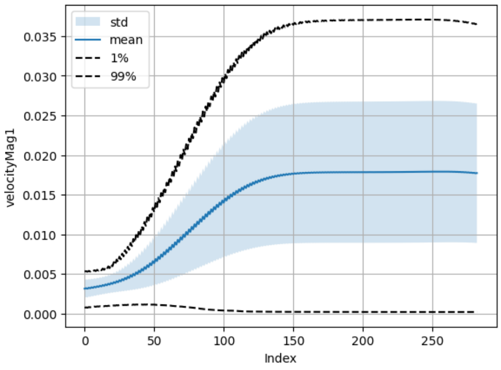

(f) Branch - Velocity distribution

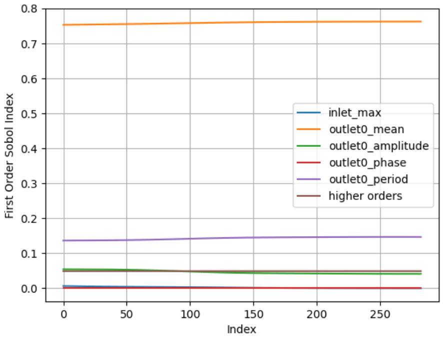

(g) Branch - Pressure Sobol indices

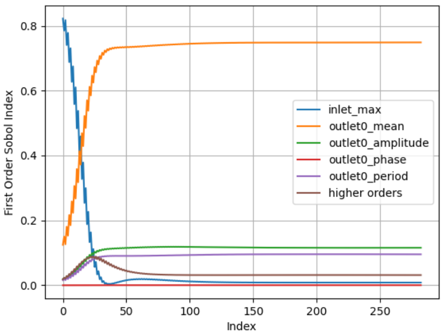

(h) Branch - Velocity Sobol indices

Supplementary Figure 18: Uncertainty analysis of the pressure and velocity within the fine bifurcation geometries determined with a third-order polynomial chaos expansion (BC1, higher pressure).

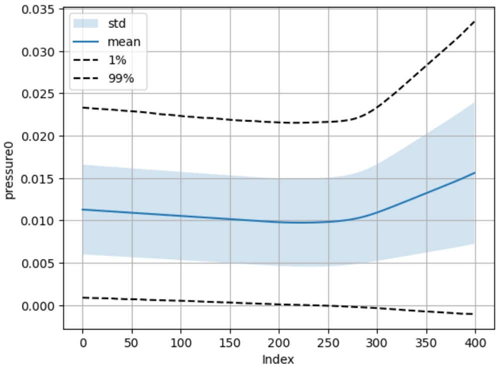

(a) Main - Pressure distribution

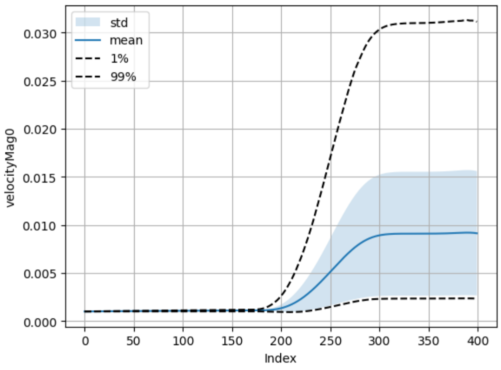

(b) Main - Velocity distribution

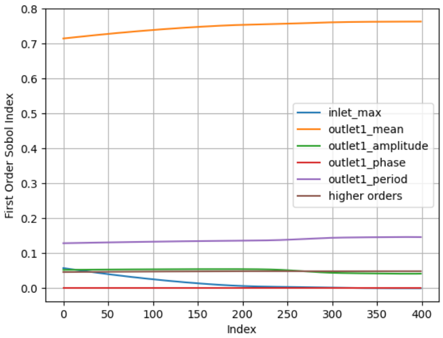

(c) Main - Pressure Sobol indices

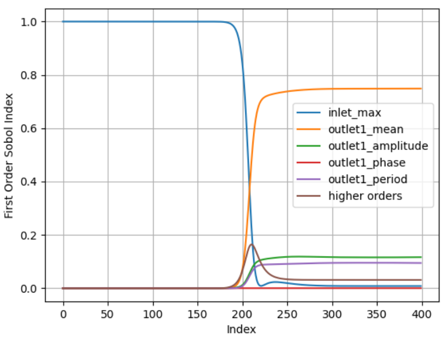

(d) Main - Velocity Sobol indices

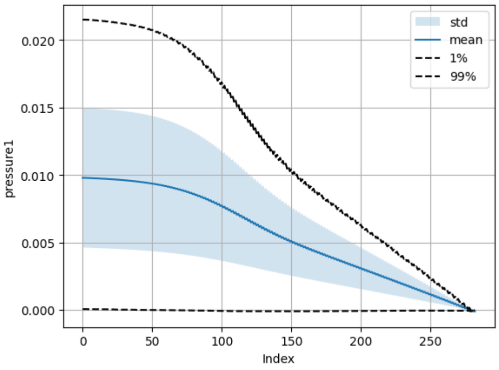

(e) Branch - Pressure distribution

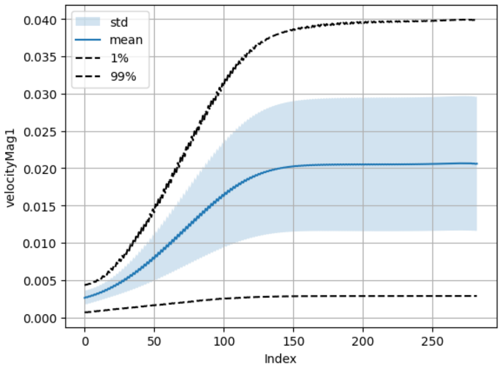

(f) Branch - Velocity distribution

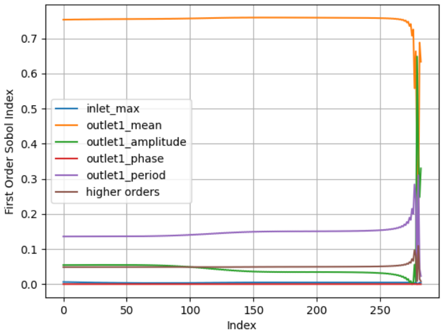

(g) Branch - Pressure Sobol indices

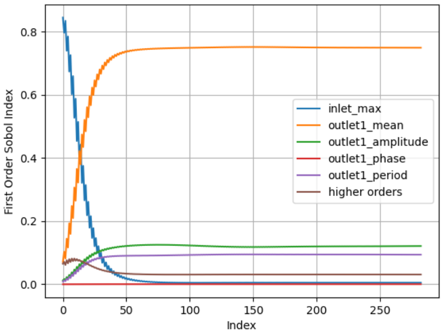

(h) Branch - Velocity Sobol indices

Supplementary Figure 19: Uncertainty analysis of the pressure and velocity within the fine bifurcation geometries determined with a third-order polynomial chaos expansion (BC2, higher pressure).

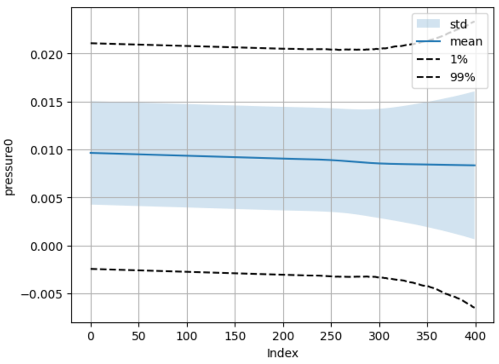

(a) Main - Pressure distribution

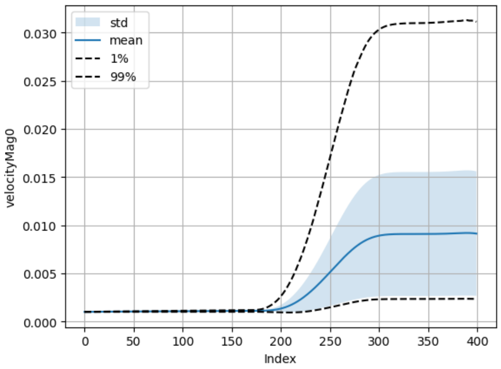

(b) Main - Velocity distribution

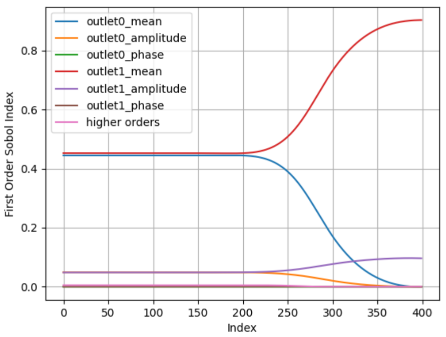

(c) Main - Pressure Sobol indices

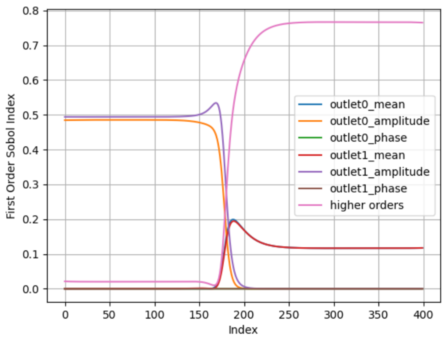

(d) Main - Velocity Sobol indices

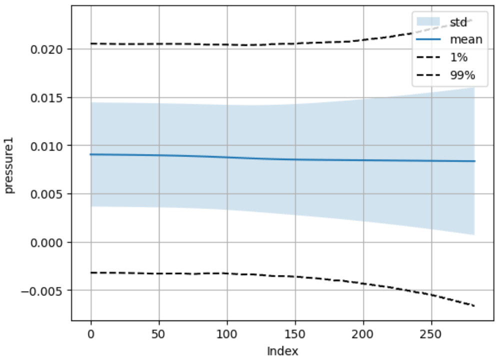

(e) Branch - Pressure distribution

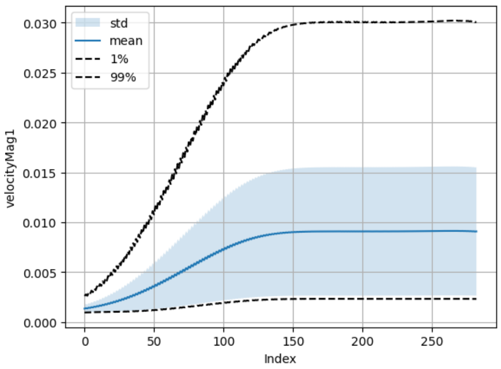

(f) Branch - Velocity distribution

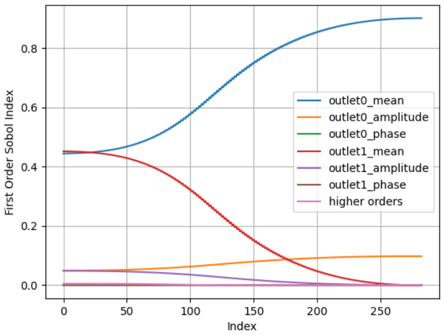

(g) Branch - Pressure Sobol indices

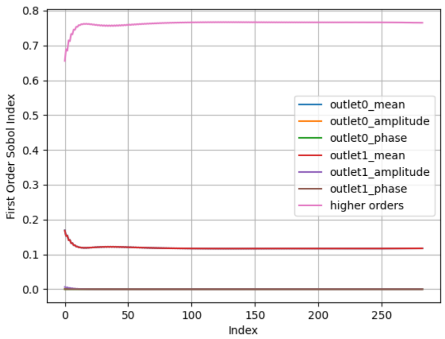

(h) Branch - Velocity Sobol indices

Supplementary Figure 20: Uncertainty analysis of the pressure and velocity within the fine bifurcation geometries determined with a third-order polynomial chaos expansion (BC3, higher pressure).

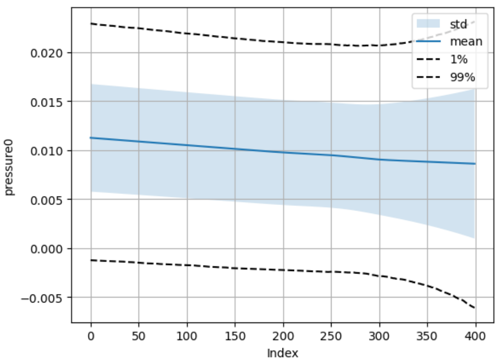

(a) Main - Pressure distribution

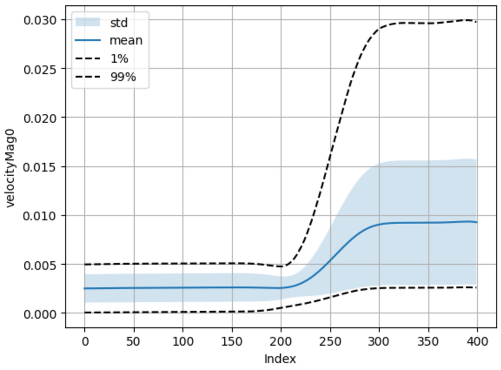

(b) Main - Velocity distribution

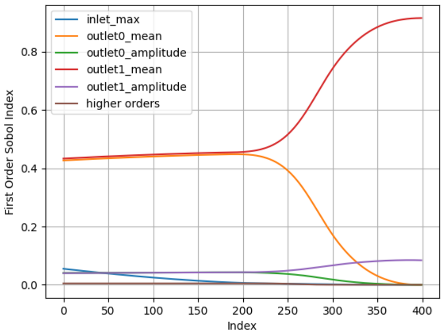

(c) Main - Pressure Sobol indices

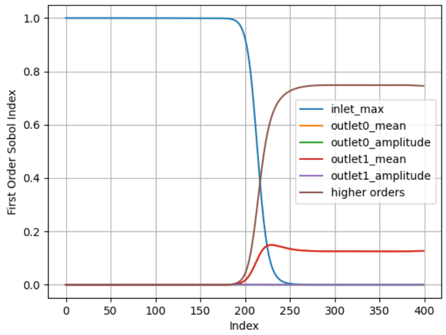

(d) Main - Velocity Sobol indices

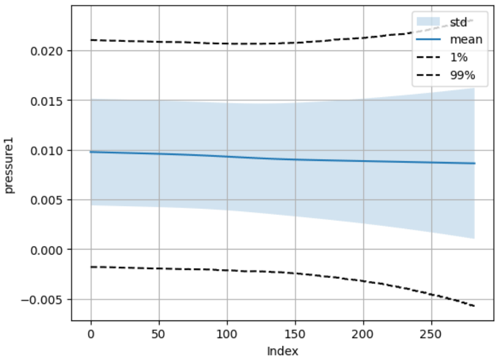

(e) Branch - Pressure distribution

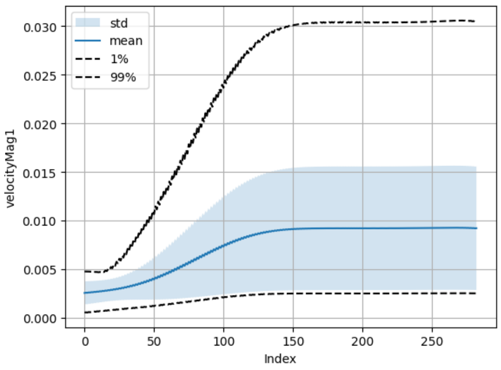

(f) Branch - Velocity distribution

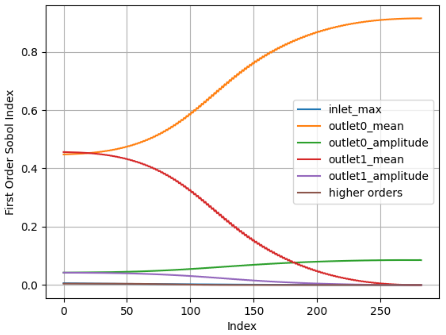

(g) Branch - Pressure Sobol indices

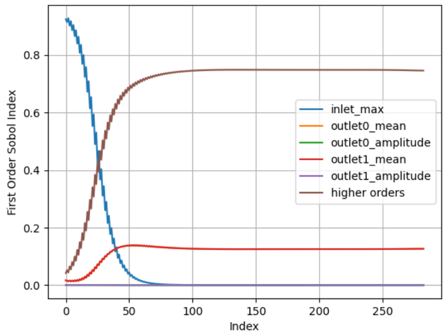

(h) Branch - Velocity Sobol indices

Supplementary Figure 21: Uncertainty analysis of the pressure and velocity within the fine bifurcation geometries determined with a third-order polynomial chaos expansion (BC4, higher pressure).

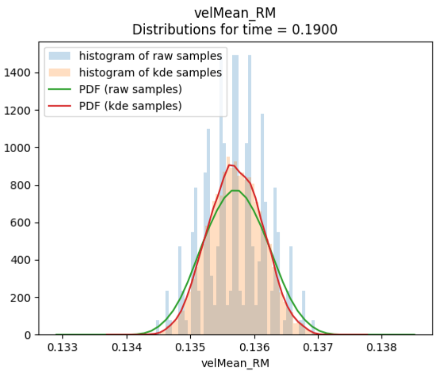

(a) Radial - Mean velocity distribution at t=0.19s

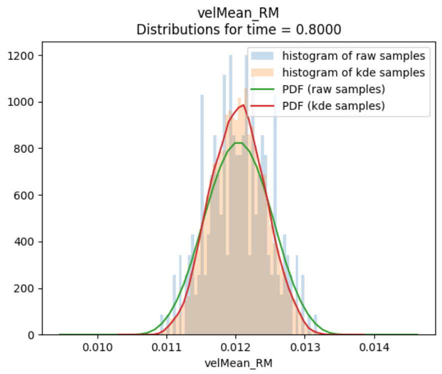

(b) Radial - Mean velocity distribution at t=0.80s

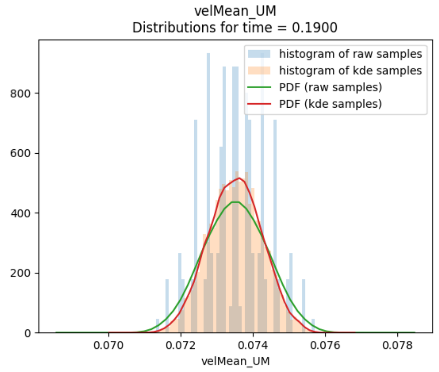

(c) Ulnar - Mean velocity distribution at t=0.19s

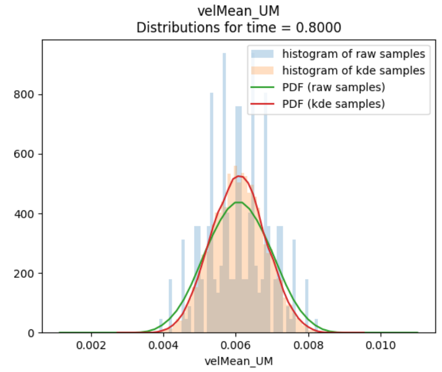

(d) Ulnar - Mean velocity distribution at t=0.80s

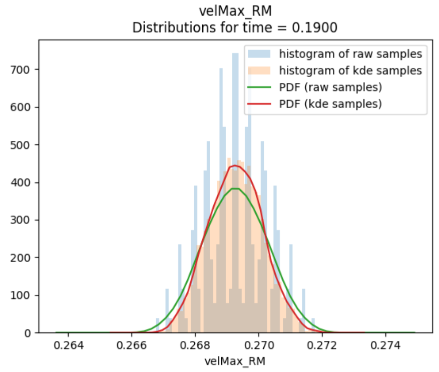

(e) Radial - Max velocity distribution at t=0.19s

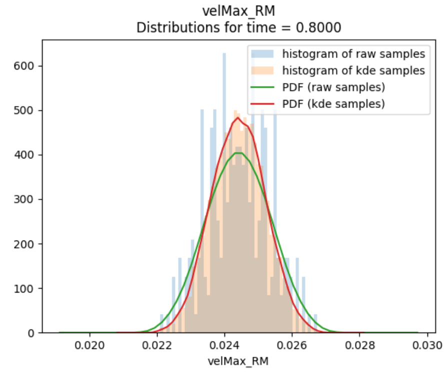

(f) Radial - Max velocity distribution at t=0.80s

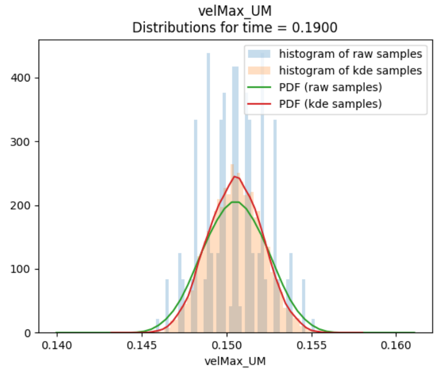

(g) Ulnar - Max velocity distribution at t=0.19s

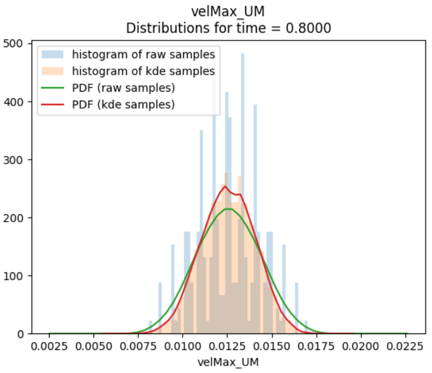

(h) Ulnar - Max velocity distribution at t=0.80s

Supplementary Figure 22: Observed distribution of the mean and maximum velocities at the analysis plane in the radial and ulnar arteries of the personalised vessels at two time points of the 0.8s heartbeat profile.
